# *STXBP6* and *B3GNT6* Genes are Associated With Selective IgA Deficiency

**DOI:** 10.3389/fgene.2021.736235

**Published:** 2021-12-17

**Authors:** Che Kang Lim, Paola G. Bronson, Jezabel Varade, Timothy W. Behrens, Lennart Hammarström

**Affiliations:** ^1^ Department of Laboratory Medicine, Karolinska Institutet, Karolinska University, Hospital Huddinge, Stockholm, Sweden; ^2^ Department Clinical Translation Research, Singapore General Hospital, Singapore, Singapore; ^3^ RED OMNI Human Genetics, Genentech, South San Francisco, CA, United States; ^4^ Biomedical Research Center (CINBIO) Singular Research Center, University of Vigo, Vigo, Spain; ^5^ Maze Therapeutics, South San Francisco, CA, United States; ^6^ Department of Biosciences and Nutrition, Karolinska Institutet, Huddinge, Sweden; ^7^ BGI-Shenzhen, Shenzhen, China

**Keywords:** immunoglobulin a deficiency, major histocompatibility complex (MHC), non-MHC genes, HLA risk allele, Stratification

## Abstract

Immunoglobulin A Deficiency (IgAD) is a polygenic primary immune deficiency, with a strong genetic association to the human leukocyte antigen (HLA) region. Previous genome-wide association studies (GWAS) have identified five non-HLA risk loci (*IFIH1, PVT1*, *ATG13-AMBRA1*, *AHI1* and *CLEC16A*). In this study, we investigated the genetic interactions between different HLA susceptibility haplotypes and non-MHC genes in IgAD. To do this, we stratified IgAD subjects and healthy controls based on HLA haplotypes (*N* = 10,993), and then performed GWAS to identify novel genetic regions contributing to IgAD susceptibility. After replicating previously published HLA risk haplotypes, we compared individuals carrying at least one HLA risk allele (HLA-B*08:01-DRB1*03:01-DQB1*02:01 or HLA-DRB1*07:01-DQB1*02:02 or HLA-DRB1*01-DQB1*05:01) with individuals lacking an HLA risk allele. Subsequently, we stratified subjects based on the susceptibility alleles/haplotypes and performed gene-based association analysis using 572,856 SNPs and 24,125 genes. A significant genome-wide association in *STXBP6* (rs4097492; *p* = 7.63 × 10^−9^) was observed in the cohort carrying at least one MHC risk allele. We also identified a significant gene-based association for *B3GNT6* (*P*_*Gene*_ = 2.1 × 10^–6^) in patients not carrying known HLA susceptibility alleles. Our findings indicate that the etiology of IgAD differs depending on the genetic background of HLA susceptibility haplotypes.

## Introduction

Immunoglobulin A deficiency (IgAD) is the most common primary immune deficiency (Yazdani et al., 2017), defined as serum levels of IgA ≤ 0.07 g/L in individuals >4 years of age who have normal serum levels of other immunoglobulins (Conley et al., 1999; Picard et al., 2015). The clinical presentation of IgAD is heterogeneous, ranging from asymptomatic blood donors to patients suffering from recurrent respiratory and gastrointestinal infections (Wang et al., 2011; Jorgensen et al., 2013). IgAD exhibits strong familial aggregation typical of a complex polygenic trait, and the strongest genetic associations have been reported in the human major histocompatibility complex (MHC) region (Ambrus et al., 1977; Cobain et al., 1983; Hammarström and Smith, 1983; MacHulla et al., 2000). The prevalence of autoimmunity, which are also strongly associated with the MHC, is strikingly higher in individuals with IgAD (Wang et al., 2011). Like autoimmune diseases, IgAD is most common in individuals of European ancestry. Thus far, two genome-wide association studies (GWAS) and one human leukocyte antigen (HLA) fine-mapping study of IgAD risk have interrogated the genetic causes of IgAD (Dostal et al., 2007; Ferreira et al., 2010; Ferreira et al., 2012; Bronson et al., 2016). However, the genetic etiology of IgAD still remains unclear.

The HLA-B*08-DRB1*03:01-DQB1*02 haplotype is the strongest genetic risk factor for IgAD in N. European populations (Olerup et al., 1990; Ferreira et al., 2012) (combined *p* = 3.37 × 10^–43^; OR = 3.33). There are two additional HLA haplotypes associated with IgAD risk: HLA-B*44-DRB1*07:01-DQB1*02 and HLA-B*14-DRB1*01:02-DQB1*05 (Olerup et al., 1990; Ferreira et al., 2012). In addition to strong linkage to the HLA region, IgAD is associated with variants in the *IFIH1, PVT1*, *ATG13-AMBRA1*, *AHI1* and *CLEC16A* gene regions (Ferreira et al., 2010; Bronson et al., 2016). Multiple studies indicated a pleiotropic effect of *IFIH1* in modulating autoimmunity, and the *IFIH1* gene region has been implicated in susceptibility to celiac disease (CD), systemic lupus erythematosus (SLE) and type 1 diabetes (T1D) (Ferreira et al., 2010; Diogo et al., 2018). Interestingly, variants in other autoimmunity genes associated with IgAD (*CLEC16A, ATG13*, and *AHI1*) are also associated with multiple sclerosis (MS) (van Luijn et al., 2015; Graves et al., 2018), though evidence for shared causal variants between IgAD and MS in these regions has not been reported.

In this study, we investigated the genetic interactions between different HLA susceptibility haplotypes and non-MHC genes in IgAD. To do this, we stratified IgAD subjects based on HLA haplotypes, and then performed genome-wide association studies (GWAS) to identify novel genetic regions contributing to IgAD susceptibility.

## Materials and Methods

### Sample Collection

In total, 806 anonymized IgAD patients and 10,187 controls were genotyped (*N* = 10,993), including 767 Swedish IgAD cases, 485 healthy Swedish controls collected from a previous study (Ferreira et al., 2012), and 9,741 Swedish twin samples (monozygotic (MZ): 4063, one per family; dizygotic (DZ): 5678, two per family) (Frankowiack et al., 2015), 39 of whom were identified with IgAD (serum IgA< 0.07 g/L). Ethical approval was obtained from the Regional ethical review board in Stockholm.

### Genotyping and Filtering

Genotyping was performed using arrays developed by Illumina, Inc. (San Diego, CA, United States). IgAD cases were genotyped on Omni1-Quad and Omni2.5 by Genentech Inc (South San Francisco, CA, United States) and the Mutation Analysis Core Facility at the Karolinska University Hospital (Stockholm, Sweden). Controls were genotyped on Omni1-Quad (Ferreira et al., 2010), except for the twin gene controls (Magnusson et al., 2013) which were genotyped on the OmniExpress. SNPs were mapped to genome build hg19 coordinates using liftOver. In addition, strand, alleles and positions were updated according to strand data mapped to hg19 (Rayner, 2011). Prior to merging datasets, we used the Genotype Harmonizer (Deelen et al., 2014) to align the format and the strands for all arrays (reference: 1000 Genomes Project Phase 3 integrated variant set). In addition, variants with evidence of deviation from Hardy-Weinberg equilibrium in the controls (*p* < 1 × 10^−6^) and a genotyping rate < 97% were removed.

### Imputation and Verification of HLA Alleles

We used a haplotype graph model to impute four-digit HLA alleles (HLA*IMP:02) (Dilthey et al., 2013), using a European reference panel and absolute posterior probability (Q2) ≥ 0.7 as a cut-off for *HLA-B, HLA-DRB1* and *HLA-DQB1*. We validated our HLA imputation by comparing it to four-digit HLA types [PCR-SSP, sequence-based typing (BST)] in 150 IgAD cases, as well as 25 healthy controls. In addition, we had two-digit HLA types for 617 cases. After verification, 636 cases and 7,798 controls with high-confidence four-digit HLA alleles were included in the analyses.

### Sub-Classification of Population Cohort and Association Analysis

We initiated the analysis by comparing individuals carrying at least one HLA risk haplotype (i.e., HLA-B*08:01-DRB1*03:01-DQB1*02:01 or HLA-DRB1*07:01-DQB1*02:02 or HLA-DRB1*01-DQB1*05:01) with individuals lacking a risk haplotype. The significantly associated variants in the sample cohort were then verified using the control cohort. Only unique variants in the cases were considered as having an association with the IgAD. We next applied the same strategy to study and verify the signal by using the cohort carrying at least one HLA-B*08:01-DRB1*03:01-DQB1*02:01 risk haplotype, the most numerous cohort (54% of total IgAD individuals). The analysis was first performed by comparing all individuals without HLA-B*08:01-DRB1*03:01-DQB1*02:01, followed by a comparison with individuals lacking all risk haplotypes.

For further analysis, cohorts homozygous for HLA-B*08:01-DRB1*03:01-DQB1*02:01 (68 IgAD and 123 controls), HLA-DRB1*07:01-DQB1*02:02 (7 IgAD and 30 controls) and HLA-DRB1*01-DQB1*05:01 (34 IgAD and 68 controls) were selected. Additionally, we also investigated cohorts homozygous for single alleles, i.e. HLA-B*08:01, HLA-DRB1*03:01, HLA-DRB1*07:01 and HLA-DQB1*05:01.

*Chi square* tests of association on genotypes for each cohort were performed independently, using only variants that overlapped between the arrays used to genotype the cohort. Variants reaching genome-wide significance (*p* < 5 × 10^−8^) were considered significantly associated with IgAD. In addition, variants with *p* < 2 × 10^−7^, and FDR ≤ 0.05 were considered to show a suggestive significant association with IgAD. In addtion, the locus zoom plots (Pruim et al., 2010) were visually inspected to confirm that the association signal is consistent with the LD pattern of the SNPs in the region. Furthermore, visualization of the linkage effect for several multi-allelic HLA types was analyzed using the Disentangler software (Kumasaka et al., 2010; Okada et al., 2011a).

### Gene-Based Association Analysis in Different Subgroups

GCTA-fastBAT analysis was performed to investigate gene-based associations (Bakshi et al., 2016). The method performs a set-based association analysis for human complex traits using summary-level data from genome-wide association studies (GWAS) and linkage disequilibrium (LD) data from a reference sample with individual-level genotypes. In total, 24,125 genes (including 1,522 miRNA genes) (hg19) were included in the analysis. Genes in the MHC region (chr6:25300000–33800000) were excluded due to strong LD in the region. The gene region was defined as + 50 kb from both 3′ and 5′ UTR of the genes. The LD cut off was set at 0.9. Assuming independence of the gene-level tests, non-MHC genes that had at least five SNPs in the region and *P*_Gene_ < 2.10 × 10^−6^ were considered significant. However, this threshold is conservative since there is overlap between genes, so 2.1 × 10^−6^ < *P*_Gene_ < 2.10 × 10^−4^ was also reported and considered to be suggestive of association.

### LD Proxy Analysis of Associated Variants

LD proxy analysis was performed using LDlink (Machiela and Chanock, 2015) (EUR; *r* ^*2*^ > 0.9 and *D'* > 0.9).

### Polygenic Risk Score Pathway Set Analysis

PRS pathway based analysis was performed using PRsice/PRSet (Choi and O'Reilly, 2019) to explore the differences of associated pathways in the cohort carrying at least one MHC susceptibility allele as compared to individuals lacking a risk haplotype. A total of 4,762 pathways genes sets from the Molecular Signatures Database (MSigDB) (Subramanian et al., 2005; Liberzon et al., 2015) were included in the analysis. The pathway sets that had at least 15 SNPs in a gene set and had a total *p* < 5 × 10^−6^ were considered significant. The prevalence of IgAD in Sweden (1: 600) was used to adjust the R^2^ (variance explained).

## Results

### HLA Association Analysis and Multiple Haplotype Interaction Investigation

8,434 samples passed QC and were included in the analysis (636 IgAD and 7,798 controls). In the single haplotype analysis, the HLA-B*08:01-DRB1*03:01-DQB1*02:01 haplotype showed the strongest association with IgAD (OR = 3.59, P_*+++*_ = 3.17 × 10^–82^), while HLA-DRB1*07:01-DRB1*02:02 (OR = 1.84, P_*++*_ = 1.19 × 10^–9^) and HLA-DRB1*01:01- DQB1*05:01 (OR = 1.41, P_*++*_ = 1.31 × 10^–4^) showed weaker associations (Table 1). The HLA-DRB1*01:02 DQB1*05:01 effect was not possible to calculate due to the low frequency in controls (F < 0.01). Hence, a combined signal of HLA-DRB1*01-DQB1*05:01 (a combination of HLA-DRB1*01:01 and HLA-DRB1*01:02) was investigated and a strong association signal was detected (OR = 1.84, P_*++*_ = 3.90 × 10^–14^). However, the presence of HLA-B*08:01 (OR = 1.32, P_*+--*_ = 1.17 × 10^–1^) or HLA-DRB1*03:01-DQB1*02:01 (OR = 1.32, *p* = 7.24 × 10^–2^) alone was not associated with IgAD susceptibility. Similarly, the presence of HLA-DRB1*07:01 (OR = 1.06, P *P*_*PA*_ = 7.16 × 10^–1^) was not associated with IgAD (Table 1). *HLA*-DQB1*02:02 is in complete LD with HLA-DRB1*07:01 and thus, we did not identify any case that allowed us to investigate the effect of *HLA*-DQB1*02:02 without the presence of HLA-DRB1*07:01. Similarly, the number of cases and controls was too low to determine the effect of *HLA*-DRB1*01 or *HLA*-DQB1*05:01 alone.

**TABLE 1:** HLA haplotype association analysis for high-risk susceptibility alleles in the IgAD cohort.

	HLA haplotypes	Allele Present (+)/Absent (−)	Freq case	Freq control	OR	P	Significance
1	HLA-B*08:01-DRB1*03:01-DQB1*02:01	+++	0.32	0.11	3.59	P_+++_ = 3.17E-82	***
	HLA-B*08:01-DRB1*03:01-DQB1*02:01	−++	0.04	0.03	1.32	P_−++_ = 7.24E-02	—
	HLA-B*08:01-DRB1*03:01-DQB1*02:01	+−−	0.02	0.02	1.17	P_+−−_ = 4.30E-01	—
2	HLA-DRB1*07:01-DQB1*02:02	++	0.10	0.05	1.84	P_++_ = 1.19E-09	***
	HLA-DRB1*07:01-DQB1*02:02	+−	0.03	0.03	1.06	P_+−_ = 7.16E-01	—
3	HLA-DRB1*01:01-DQB1*05:01	++	0.12	0.09	1.41	P_++_ = 1.31E-04	**
	HLA-DRB1*01:01-DQB1*05:01	-+	0.05	0.01	3.35	P_−+_ = 2.24E-16	***
	HLA-DRB1*01:02-DQB1*05:01	−+	0.13	0.10	1.38	P_−+_ = 2.25E-04	**
	HLA-DRB1*01-DQB1*05:01	++	0.16	0.09	1.84	P_++_ = 3.90E-14	***

Only a frequency of > 0.01 in both cohorts were included in the analysis.

*P*: Significance level * <0.01; **<0.001; ***< 0.0001.

**FIGURE 1:**
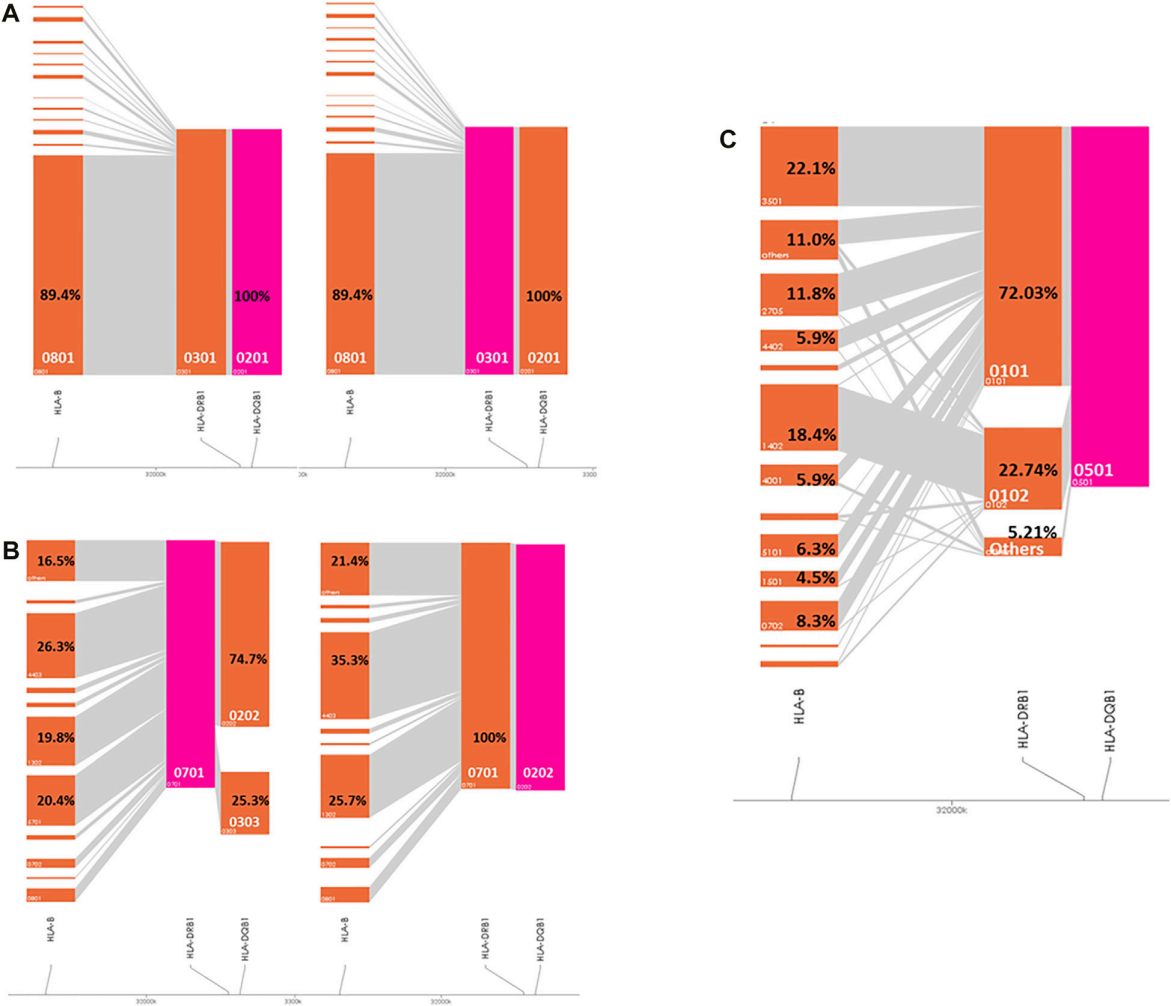
Visualization of the HLA haplotype structure in IgAD patients. Pink color marks the investigated haplotype. **(A)** HLA-B*08:01-DRB1*03:01-DRB1*02:01 cohort; **(B)** HLA-DRB1*07:01-DQB1*02:02 cohort; **(C)** HLA-DRB1*01-DQB1*05:01 cohort.

As shown in Figure 1A, in the IgAD patients, HLA-DQB1*02:01 and HLA-DRB1*03:01 are in perfect LD. HLA-DQB1*02:02 and HLA-DRB1*07:01 are also in perfect LD. HLA-DRB1*07:01 occurred with HLA-DQB1*02:02 in 74.7% of the analyzed individuals and with HLA-DQB1*03:03 in 24.7% of individuals (Figure 1B). 72% of the HLA-DRB1*01:01 alleles and 22.8% of the HLA-DRB1*01:02 alleles occurred with HLA-DQB1*05:01, whereas the remaining 5.2% mainly occur with HLA-DRB1*01:03 and DRB1*10:01 (Figure 1C).

### Analysis of the Influence of Non-MHC Variants in IgAD Patients Homozygous for High-Risk HLA Allele

Based on the above haplotype and linkage analyses, we subdivided the patients into HLA susceptibility groups to investigate non-MHC gene interactions with the known susceptibility haplotypes.

**FIGURE 2:**
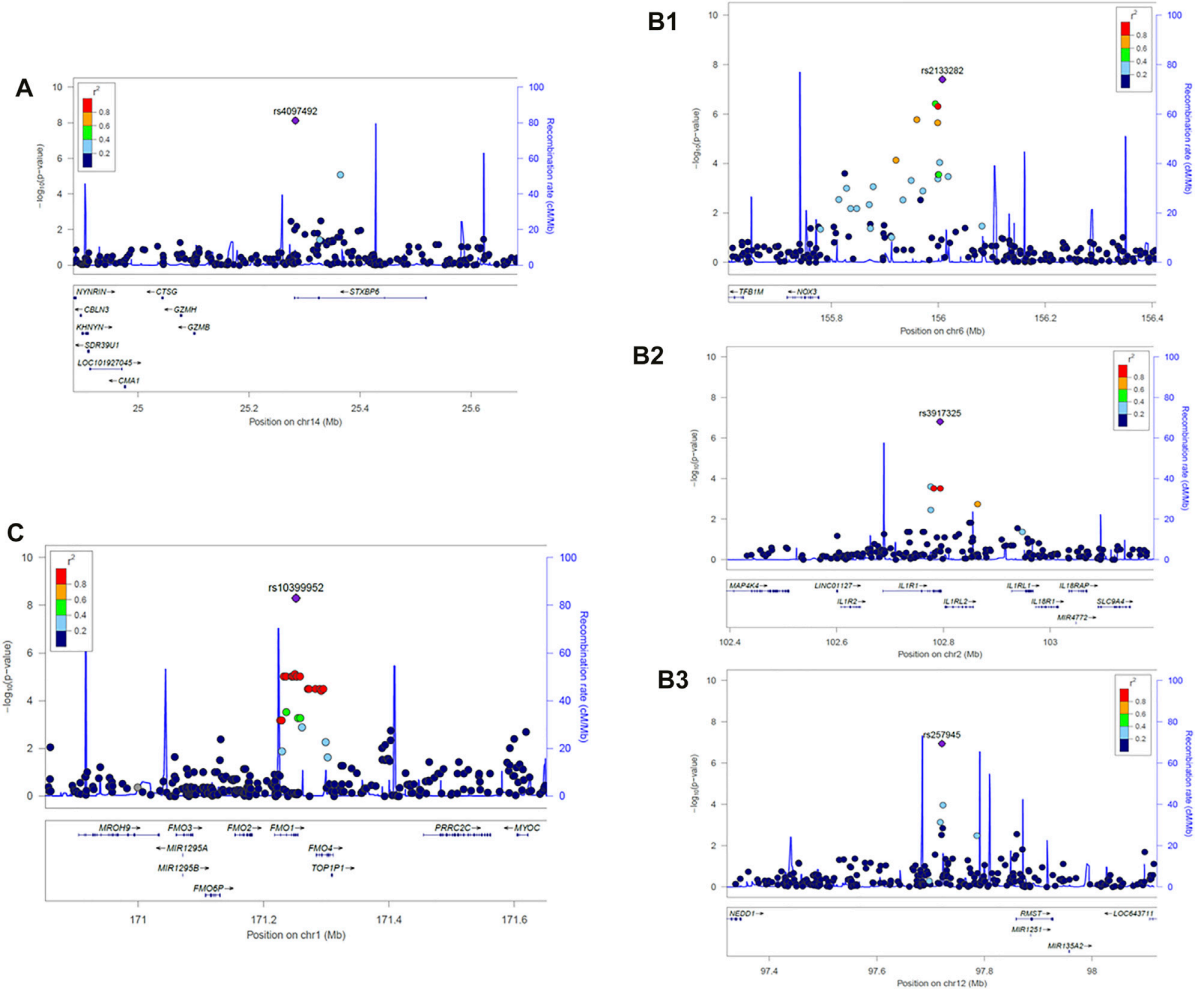
LocusZoom plot of the associated loci in IgAD patients carrying respective MHC susceptibility haplotypes. **(A)** Carrying at least one MHC risk haplotype (HLA-B*08:01-DRB1*03:01-DQB1*02:01 or HLA-DRB1*07:01-DQB1*02:02 or HLA-DRB1*01-DQB1*05:01), rs4097492, Chr14:25283341 **(B)** HLA-DRB1*07:01-DQB1*02:02 homozygous cohort, **(B1)**: rs2133282, Chr6:156007516, **(B2)**: rs3917325, Chr2: 102793907, **(B3)**: rs257945, Chr12:97720902; **(C)** HLA-DRB1*01-DQB1*05:01 homozygous cohort, rs10399952, Chr1:171251663. The associated locus (top SNP) is represented by purple circle in each LocusZoom plot. All the other SNPs are colored based on their correlation (r^2^) with the labeled top SNP. The recombination rates estimated from 1,000 Genomes (EUR) data are shown in solid blue line. Genes are marked below by horizontal blue lines and the arrows on the horizontal blue lines show the direction of transcription. Gene designations and physical positions and are based on the Genome Reference Consortium Human Build 37, GRCh37.

We first analyzed the difference between individuals carrying at least one HLA risk allele with individuals lacking any risk allele. Based on the cross-comparison strategy (Supplementary Figure S1), we identified one significantly associated non-MHC variant, rs4097492 (OR = 0.23, *p* = 7.63 × 10^–9^), an intronic variant of the *STXBP6* gene on chromosome 14 (Figure 2A). Next, we compared individuals carrying at least one HLA-B*08:01-DRB1*03:01-DQB1*02:01 haplotype to individuals who lack the HLA-B*08:01-DRB1*03:01-DQB1*02:01 haplotype (but including individuals carrying another risk haplotypes). There was no significant association.

In the HLA-DRB1*07:01-DQB1*02:02 homozygous cohorts, one significant (<5 × 10^–8^) and two strongly suggestive variants (<2 × 10^–7^; FDR ≤ 0.05) were identified (Figure 2B1). The peak variant was rs2133282 (OR = 33, *p* = 3.97 × 10^–8^; FDR = 0.02), an intergenic variant located between the *NADPH* oxidase *NOX3* and the tumor suppressor *ARID1B* on chromosome 6. The two novel strong suggestive variants were rs3917325 (OR = 59, *p* = 1.57 × 10^–7^; FDR< 0.05), an UTR3 variant of *IL1R1* on chromosome 2 and rs257945 (OR = 38.67, *p* = 1.14 × 10^–7^; FDR = 0.05), an intergenic variant located between *NEDD1* and *RMST* on chromosome 12. For the HLA-DRB1*07:01 single allele homozygous cohort, a UTR3 variant of the Interleukin Receptor 1 (*IL1R1*) on chromosome 2 (rs3917325) was significantly associated (OR = 21.83, *p* = 3.55 × 10^–8^; FDR = 0.01) (Figure 2B2).

For the HLA-DRB1*01-DQB1*05:01 homozygous individuals, one significant marker, rs10399952 (OR = 15.4, *p* = 5.05 × 10^−9^), a variant of microsomal flavin-containing monooxygenase 1(*FMO1*) on chromosome 1, was detected (Figure 2C). We did not detect any strong signal (<5 × 10^–8^ or < 2 × 10^–7^) in the HLA-B*08:01-DRB1*03:01-DQB1*02:01 homozygous cohort based on this analysis method (Supplementary Figure S2D), nor in the single allele homozygous cohorts carrying HLA-B*08:01, DRB1*03:01 or DQB1*05:01 (Supplementary Figure S2E–G).

### Analysis of Influence Genes Using Gene-Based Analysis in Different Subgroups

The effect sizes of individual genetic variants are usually small because of the polygenic nature of human complex traits and diseases which limits the statistical power to detect them. Emerging evidence has suggested that diseases or traits associated variants identified in genome-wide association studies (GWAS) tend to be located in gene-rich regions (Yang et al., 2011; Schork et al., 2013). SNPs in and around genes have been shown to explain more phenotypic variation (Yang et al., 2011) and tend to have enriched replicable associations at higher rates (Smith et al., 2011) than intergenic SNPs. In addition, multiple associated variants at a single locus are generally observed (Wood et al., 2014). Therefore, for the investigation of complex trait genes, it is more powerful to test the aggregated effect of a set of SNPs within a gene region.

To enhance our detection power, we analyzed the data using a gene-based association analysis. As HLA is co-dominantly expressed, only patients homozygous for the susceptibility HLA haplotypes were included in the analysis. The HLA-DRB1*07:01 HLA-DQB1*02:02 cohort was excluded due to the small number of cases (<20). In addition, we also tested patient cohorts carrying no HLA susceptibility alleles.

With the enhanced method, *CD40* (*p* = 6.9 × 10^–5^), with a total of 29 SNPs in the analyzed region, was found to be suggestively associated with IgAD patients homozygous for the HLA-B*08:01-DRB1*03:01-DQB1*02:01 haplotype. *DHX38* (*p* = 8.6 × 10^–5^) and 14 SNPs in a region containing a novel inhibitor of protein phosphatase 4 (Han et al., 2015), showed suggestive evidence for association in patients homozygous for HLA-DRB1*01-DQB1*05:01.

**FIGURE 3:**
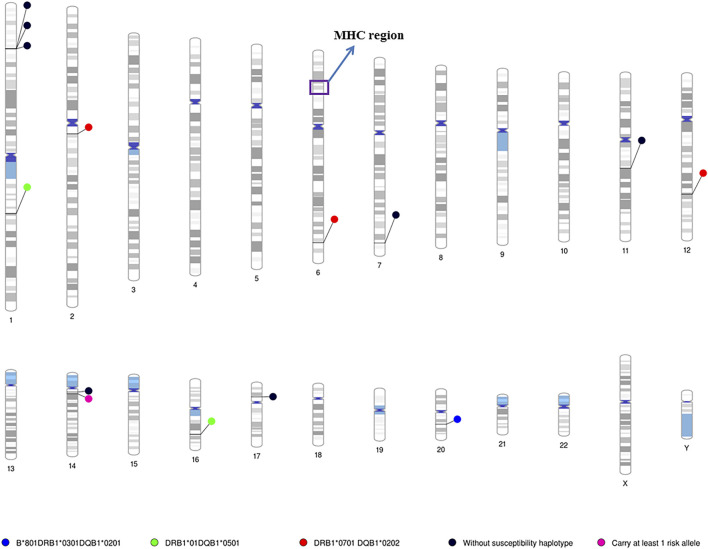
Chromosome ideograms for all identified susceptibility genes/loci in the patients with different HLA risk alleles. Chromosome ideograms were generated using the Phenogram software (Wolfe et al., 2013). Blue circle: location of genes/locus associated with the HLA-B*08:01-DRB1*03:01-DQB1*02:01 homozygous cohort; Green circle: location of genes/locus associated with the HLA-DRB1*01-DQB1*05:01 homozygous cohorts; Red circle: location of genes/locus associated with the HLA-DRB1*07:01-DQB1*02:02 homozygous cohorts; Black circle: location of genes/locus associated with patients do not carry any MHC susceptibility haplotye. Pink circle: location of genes/locus associated with patients carrying at least one MHC susceptibility genes. The MHC region is highlighted in purple box. Coloured regions indicated the cytogenetic band on each chromosome according to the predefined setting of the Phenogram, which is based on ideogram documented in the UCSC database (Furey and Haussler, 2003).

In patients who do not carry any of the major HLA susceptibility haplotypes, we identified a significant association with *B3GNT6* (*p* = 2.1 × 10^–6^), which encodes an important precursor in the biosynthesis of mucin-type glycoproteins (Supplementary Table S1). In addition, another region containing six genes showed suggestive evidence for association with IgAD. Many of the genes in this region have been associated with autoimmune diseases or DNA repair (Supplementary Table S1). The locations of all the identified genes from two different methods are shown in Figure 3 according to the HLA susceptibility groups.

### Linkage Disequilibrium Proxy Analysis

Ldlink analysis was performed to explore proxy and putatively functional variants within the nearby genetic region that correlated with the associated variants. Rs4097492 proxies are clustered close to the *STXBP6* genes (Supplementary Figure S4A). Supplementary Figure S4B shows that rs213382 proxies are clustering near the *NOX3* gene. Based on the results, rs213382 is not linked with the nearby *mir1202* gene. On the other hand, rs3917325 proxies cluster around the *IL1R1* and *IL1RL2* gene regions (Supplementary Figure S4C). Similarly, rs10399952 proxies cluster close to the *FMO1* and *FMO4* genes (Supplementary Figure S4E); additionally, rs10399952 is an eQTL of FMO4 (Ex Portal (2021).Ex, 2021). On the other hand, rs257945 proxies are close to the *RMST* gene but are not in strong LD with the nearby *mir1251* and *mir135A2* microRNAs (Supplementary Figure S4D).

### Polygenic Risk Score Pathway Set Analysis

**FIGURE 4:**
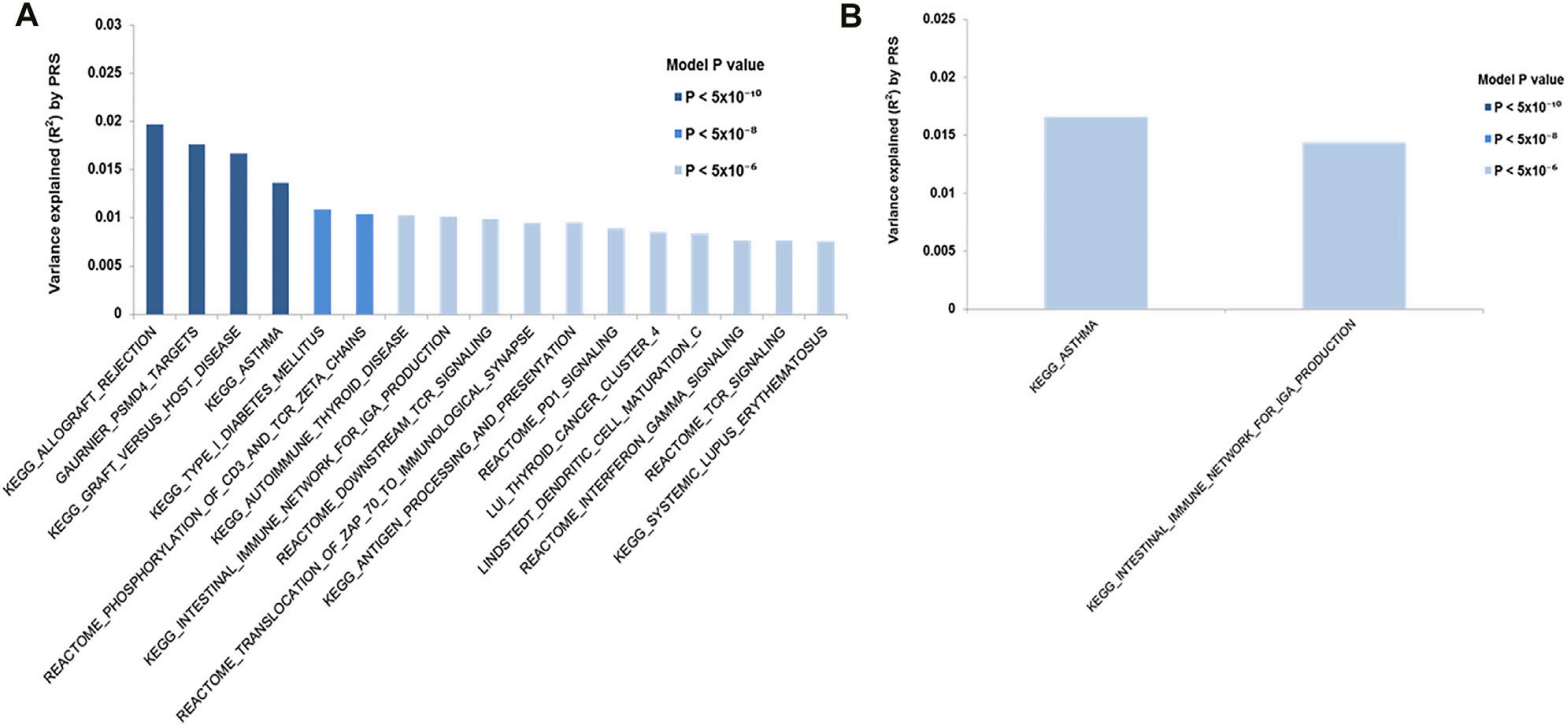
PRS (Polygenic risk score) pathways/gene sets analysis. PRS pathway/gene set analysis for IgAD cohort carrying different MHC risk haplotypes. Only significant pathway/genes set (*p* < 2 × 10^−6^) are shown. The y-axis indicates R (Conley et al., 1999), a measure of the variance explained. On the x-axis showing the significant pathways/gene sets. The color of the bar indicate the *p* value threshold. **(A)** HLA-B*08:01-DRB1*03:01-DRB1*02:01 cohort; **(B)** HLA-DRB1*01-DQB1*05:01

PRSet pathway/gene set based analysis was performed to explore the pathway association in each cohort carrying MHC risk allele using the individuals lacking all risk haplotypes as a reference. The analysis results suggested that individuals carrying HLA-B*08:01-DRB1*03:01-DQB1*02:01 risk haplotypes were enriched for association with various immune conditions e.g., allergy, asthma, autoimmunity (e.g., SLE, T1D) (Figure 4A). Two significant pathway sets, i.e. asthma and intestinal immune network for IgA production, were detected in the cohorts carrying the HLA-DRB1*01-DQB1*05:01 risk haplotypes (Figure 4B). However, no significant pathway sets (*p* < 2 × 10^−6^) were identified in the cohort carrying *HLA*-DRB1*07:01-DQB1*02:02.

## Discussion

MHC risk haplotypes for IgAD are neither fully penetrant nor required for disease, however, it is not yet known whether biological interaction between an MHC susceptibility allele and a non-MHC susceptibility allele contributes to disease onset.

HLA was first described as a risk locus for IgAD through the association with classical HLA class I and class II alleles (Ambrus et al., 1977; Cobain et al., 1983; Hammarström and Smith, 1983), and the extended HLA-A*01-B*08-DRB1*03-DQB1*02 haplotype has been identified as the strongest genetic risk factor for IgAD in N. European populations (Olerup et al., 1990). In addition, significant associations with two other haplotypes, DRB1*01:02-DQB1*05:01 and DRB1*07:01-DQB1*02, have been reported (Olerup et al., 1990; Ferreira et al., 2012). Nonetheless, the causal HLA risk allele(s) have not yet been identified.

Interaction between an HLA allele and a non-MHC genetic variant has been suggested in several autoimmune diseases, including T1D (Bjørnvold et al., 2006; Steck et al., 2006; Smyth et al., 2008), SLE (Bjørnvold et al., 2006), Graves Disease (GD) (Hodge et al., 2006; Kula et al., 2006; Jacobson et al., 2008; Takahashi and Kimura, 2010) and Myasthenia gravis (MG) (Varade et al., 2017). However, to date, it has not been described in IgAD. In this study, potential HLA/non-MHC interactions were investigated. In addition, we also assessed the previously identified association signal in autoimmune diseases and IgAD according to HLA risk alleles (Supplementary Table S1). In total, 14 novel genes/loci (4 significant and 10 suggestive) were identified in patients carrying different HLA susceptibility haplotypes (Figure 3). The majority have been implicated in immune function and autoimmune diseases. However, none of them have been reported to modify risk of HLA alleles in autoimmune diseases.

Our results show that in patients who carry at least one HLA risk haplotype, a common genetic variation in an intronic region of Syntaxin Binding Protein 6 (*STXBP6*) was significantly associated with protection against IgAD. This finding suggests that individuals who carry at least one risk haplotype and who do not carry the protective *STXBP6* allele have a higher risk of developing disease. The signal remained significant when we compared individuals carrying at least one HLA-B*08:01-DRB1*03:01-DQB1*02:01 haplotype to individuals lacking a risk allele. However, when we performed a comparison of individuals carrying at least one HLA-B*08:01-DRB1*03:01-DQB1*02:01 allele with individuals lacking HLA-B*08:01-DRB1*03:01-DQB1*02:01 only (individuals carrying another risk haplotype were included), the signal was weaker (*p* = 7.00 × 10^−4^). The observation may be due to the lack of an added effect from the other two risk haplotypes. When we compared individual carrying at least one HLA-DRB1*07:01-DQB1*02:02 or HLA-DRB1*01-DQB1*05:01 haplotype with individuals lacking a risk allele, there was no genome-wide signal (*p* < 5.00 × 10^–8^) and the signal at rs4097492 was weaker (OR = 0.31, *p* = 5.13 × 10^–5^) due to the small cohorts of the other two risk haplotypes. This observation suggests that it is a modest association signal for individual carrying HLA-DRB1*07:01-DQB1*02:02 or HLA-DRB1*01-DQB1*05:01 risk haplotype.

*STXBP6*, a gene associated with white blood cell counts (Kichaev et al., 2019; Okada et al., 2011b), contains a phosphatidylinositol 4,5-bisphosphate (PIP2) binding domain (http://www.ebi.ac.uk/interpro/entry/ InterPro/ IPR028258/) and has been reported to play a role in regulating soluble N-ethylmaleimide-sensitive factor attachment protein receptor (SNARE) complex formation (Scales et al., 2002). PIP2 is in the PI3K signaling pathway, and rare mutations in PIK3R1 (involved in the phosphorylation of PIP2 to PIP3) are associated with an immune deficiency that includes lack of IgA production. (Walsh and Fruman, 2014). In the STRING database (Jensen et al., 2009), *STXBP6* is associated with SNARE proteins, including *STX4, SNAP25, STXBP5* (Supplementary Figure S3). One of these SNARE proteins, *STX4,* appears to play an essential role in the secretion of antibodies by human plasma cells (Gómez-Jaramillo et al., 2014). In addition, the expression of *STXBP6* (http://www.proteinatlas.org) (Uhlén et al., 2015) is enriched in gamma delta (γδ) T cells (Blood Atlas. Blood Atlas, 2020). γδ T cells express a unique T-cell receptor (TCR) composed of one γ-chain as well as one δ-chain. γδ T cells are involved in the initiation of immune responses. Generally found in low frequency in the body, γδ T cells are most abundant at mucosal surfaces such as the gut, skin, and lungs. It is thus possible *STXBP6* may be indirectly implicated in IgA secretion.

For the patients homozygous for HLA-B*08:01-DRB1*03:01-DQB1*02:01, *CD40* showed suggestive evidence of association with IgAD. *CD40* is a transmembrane receptor which belongs to the TNF receptor superfamily and is expressed on B cells, monocytes and dendritic cells (Paulie et al., 1985). It is a crucial player in both innate and adaptive immune responses and involved in the regulation of humoral immunity and cytokine production. Decreased expression of *CD40* on monocytes of children with IgAD has previously been observed (Kowalczyk et al., 2006) and *CD40* has also been implicated in the etiology of a variety of immune diseases such as RA, asthma, T1D and MS (Park et al., 2007; Australia and New Zealand, 2009; van der Linden et al., 2009).

The DEAH-box helicase 38 (*DHX38*) gene and rs10399952, a variant in *FMO1*gene was associated with the development of IgAD in patients homozygous for HLA-DRB1*01-DQB1*05:01. rs10399952 is an eQTL of *FMO4* (Ex Portal (2021).Ex, 2021), *FMO4* is one of the significantly differentially expressed genes identified in the galactose-deficient IgA inducing mesangial cells (Liu et al., 2017). *DHX38* is an RNA helicase, involved in the alteration of RNA secondary structure such as translation initiation as well as ribosome and spliceosome assembly (Schwer and Guthrie, 1991; Wang and Guthrie, 1998; Hegele et al., 2012). Recent studies have suggested that it is a novel inhibitor of protein phosphatase 4 (*PP4*) (Han et al., 2015). As *PP4* is essential for the germinal center formation and class switch recombination in mice (Chen et al., 2014), suggesting that *DHX38* may be involved in the development of IgAD in the HLA-DRB1*01-DQB1*05:01 subgroup.

We identified a UTR variant, rs3917325, (MAF: 0.038) in *IL1R1* associated with IgAD in the HLA-DRB1*07:01-DQB1*02:02 cohort. Interestingly, LD pair testing, using the EUR population in the 1000G genome cohort, shows that the variant is in linkage with rs10490571 (D’:0.95, R^2^:0.03), a locus reported as being associated with Immunoglobulin A nephropathy (IgAN) (Xie et al., 2017), a disease which is linked to overproduction of IgA. This observation suggests that *IL1R1* may potentially play a role in IgA production.

The cohorts lacking any HLA susceptibility alleles comprise 18.6% (118 out of 636) of the total number of patients. From gene-based association analysis in IgAD patients who do not carry the HLA risk haplotypes, we identified a significant association with *B3GNT6*, a precursor in the biosynthesis of mucin-type glycoproteins. *B3GNT6* has previously been reported to be associated with inflammatory colitis (An et al., 2007).

The clinical presentation of IgAD varies, ranging from asymptomatic “healthy” blood donors to symptomatic patients, supporting our observation of heterogeneity in the non-MHC association in individuals with IgAD depending on the HLA risk haplotypes. Further research is warranted to replicate our results, which may open up interesting perspectives for future research.

We further tested the differences between the stratified cohort using the PRS pathway/gene set analysis. The result show that, as compared to the cohorts lacking any susceptibility alleles, cohorts carrying the HLA-B*08:01-DRB1*03:01-DQB1*02:01 risk haplotype had a strong association with immune pathways including interferon-gamma signalling, TCR signalling, PD1 signaling, antigen processing and presentation as well as various immune diseases such as asthma, SLE and T1D. As IgAD has been suggested to be associated with risk for these autoimmune disorders (Wang et al., 2011) and asthma (Urm et al., 2013), our observation suggest a potentially shared common genetic regulatory pathway in the cohorts carrying the HLA-B*08:01-DRB1*03:01-DQB1*02:01 risk haplotype. Similarly, cohorts carrying the HLA-DRB1*01-DQB1*05:01 risk haplotype may potentially have a shared genetic/pathway with asthma and intestinal immune diseases.

*IFIH1* has been found to be associated with the development of IgAD in previous studies (Bronson et al., 2016; Ferreira et al., 2010). However, we did not detect an association signal in either the cohorts carrying homozygous MHC risk haplotypes, nor in those who do not carry any MHC risk haplotype. Since a compound heterozygous MHC risk haplotype effect has been described in SLE and RA (Shimane et al., 2013; Graham et al., 2007; Agrawal et al., 1995), further investigation in larger patient groups, including cohorts carrying compound heterozygous MHC risk alleles together with all homozygous cohorts was performed. A modest association signal in the *IFIH1* gene (PGene = 2.15 × 10^–4^) (Supplementary Table S2) was observed using this strategy, suggesting that the *IFIH1* association is relatively minor and potentially associated with IgAD patients carrying two MHC risk haplotypes. We also investigated the genes previously implicated in the susceptibility to IgAD, including *AHI1, ATG13-AMBRA1, CLEC16A,* mir-6891 and *PVT1* (Bronson et al., 2016; Chitnis et al., 2017). However, they did not pass the suggested significance threshold (*p <* 2.1 × 10^−4^).

The limitations of our study include a modest sample size for the disease cohort. The restriction has prevented us to split the data into discovery set and replication set. Hence, an independent replication cohort with similar power cannot be performed using the current collected samples. However, a random disease sub-cohort test has shown that the identified peak SNP, rs4097492 is the only point that has at least a suggestive significant *p*-value in all ten random samples tests (*p* = 1.72 × 10^−7^ - *p* = 9.60 × 10^−10^ for 400 random IgAD samples and *p* = 1.05 × 10^−7^ - *p* = 1.22 × 10^−10^ for 500 random IgAD samples). Additionally, a 10^8^ permutations test show that the peak SNP, rs 4097492 has an empirical *p*-value of 8 × 10^−8^ (Supplementary Table S3). Furthermore, functional network analysis suggests that the *STXBP6* may be indirectly implicated in IgA secretion. All the approaches have reassured us of the robustness of our findings. Nonetheless, further research with well-powered independent cohort is warranted to replicate our results. In addition, further increase in the sample size may help unravel more modest association signals.

In summary, we have identified multiple new susceptibility genes/variants for IgAD and shown that the pathogenesis of IgAD may differ depending on the presence of selected HLA susceptibility haplotypes. This may be potentially due to the interaction of non-MHC genes with the selected HLA susceptibility haplotypes. Further work is required to validate the novel associations and investigation of the regulatory role of associated variants through functional studies, including studies on the protein-protein interaction of HLA and non-MHC genes. Understanding the interaction/epistatic interaction between HLA and non-MHC genes may ultimately help us better understand the etiology of IgAD.

## Data Availability

The data is deposited in the European Variation Archive (EVA), the project accession number is PRJEB49292.
