# Supplementary material for: STXBP6 and B3GNT6 Genes are Associated With Selective IgA Deficiency
Source: Front Genet. 2021 Dec 17;12:736235. doi: 10.3389/fgene.2021.736235 (PMC8718598; doi:10.3389/fgene.2021.736235)
Supplement: Supplementary file 1 [file DataSheet1.docx]

**Common genetic variants in *STXBP6* and *B3GNT6* are associated with selective IgA deficiency**

### ****Lim et al.****

### ****Supplemental Data INFORMATION****

Supplemental data include five figures and two tables.

**Supplemental Figure**

Supplementary Figure 1: Comparison of Individuals with at least one susceptibility HLA haplotypes vs. individuals without susceptibility MHC haplotypes

Supplementary Figure 2: Manhattan Plot of the whole genome association analysis outside of the HLA region in IgAD patients carrying homozygous MHC risk alleles

Supplementary Figure 3: Protein network analysis of *STXBP6* using the STRING database

Supplementary Figure 4: Visualization of the HLA haplotype structures in Swedish controls and IgAD patients

**Supplemental Table**

Supplementary Table 1: Gene-based analysis results in selected autoimmune diseases and IgAD associated genes in patients carrying different MHC risk alleles

Supplementary Table 2: Gene based analysis results in selected autoimmune diseases and IgAD associated genes in patients carrying different MHC risk alleles

Supplementary Table 3. Random IgAD disease cohort analysis and Permutations test in patients carrying at least one MHC risk alleles

**SUPPLEMENTARY Figure Legends**

**Supplementary Figure 1. Comparison of Individuals with at least one susceptibility MHC haplotypes Vs Individuals without susceptibility MHC haplotypes**

Venn diagram of comparison of individuals carrying at least one susceptibility MHC alleles with individuals lacking a risk allele. The numbers indicate total variants passing the genome-wide cutoff (P < 5 x 10^-8^).

**Supplementary Figure 2. Manhattan Plot of the whole genome association analysis outside of the HLA region in IgAD patients carrying homozygous MHC risk alleles**

Red line marks the p-value cut-off. Blue line marks the suggestive association cut off. **a.** Carrying at least one MHC risk haplotype (*HLA*B0801-DRB1*0301-DQB1*0201* or *HLA-DRB1*0701-DQB1*0202* or *HLA-DRB1*01-DQB1*0501*) **b.** *HLA-DRB1*0701- DQB1*0202* cohort; **c**. *HLA-DRB1*01-DQB1*0501*;**d**. *HLA-B*08:01-DRB1*03:01-DQB1*02:01* homozygous cohort; **e**. *HLA-DRB1*08:01* single allele homozygous; **f**. *HLA-DRB1*03:01* single allele homozygous; **g**. *HLA-DRB1*05:01* single allele homozygous.

**Supplementary Figure 3. Protein network analysis of *STXBP6* using STRING database**

The node protein STXBP6 is highlighted in red and indicated by red arrow. The analysis limited to the first shell/level of interactors

**Supplementary Figure 4. Linkage disequilibrium proxy analysis**

The associated SNP is indicated by a red arrow. a.rs4097492; b. rs2133282; c. rs3917325; d.rs257945; e.rs10399952;

**Supplementary Figure 5. Visualization of the HLA haplotype structures in Swedish Controls and IgAD patients**

The vertically stacked bars represent the HLA genes (HLA-B, HLA-DRB1 and HLA-DQB1). The positions of the bars show the physical order of the genes (reference: hg19). The thickness of each segment corresponds to the frequencies of the respective HLA allele and haplotype whereas allele frequency > 2.5% are indicated. Each allele was present in 4 digit resolution.

**Supplemental Data**

**Supplementary Figure 1.**


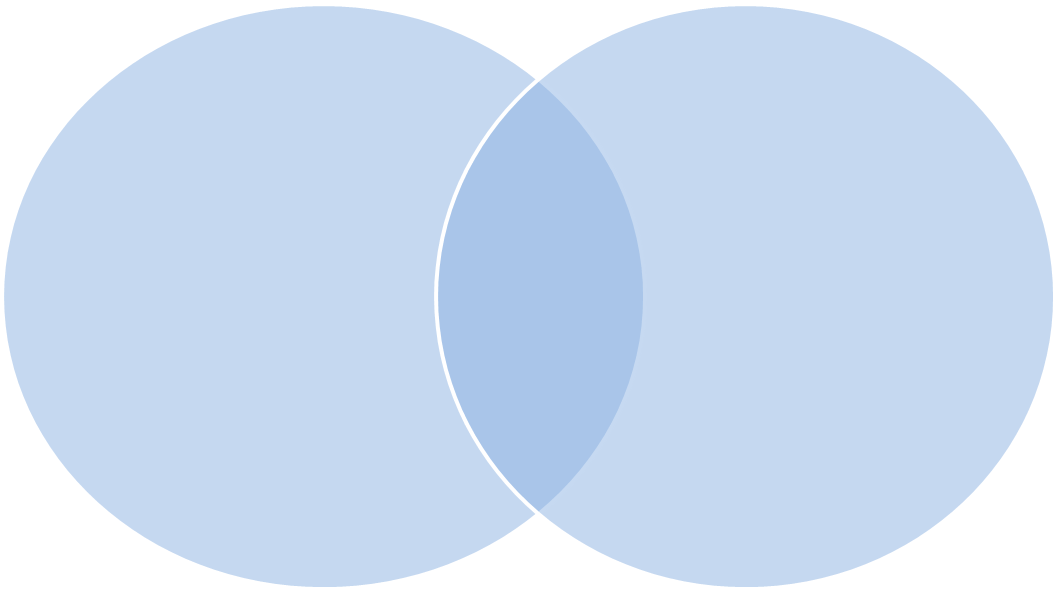


**Case**

**Control**

**With_at_least_1_MHC susceptibility alleles**

**Vs**

**without any MHC susceptibiliy**

**With_at_least_1_MHC susceptibility alleles**

**Vs**

**without any MHC susceptibiliy**

**Significant Non-MHC variants only (P< 5x 10e-8)**

**1**

**0**

**4 +**

**31 (upstream of MHC)**

**+ 7 (downstream of MHC**

**Supplementary Figure 2a**

**
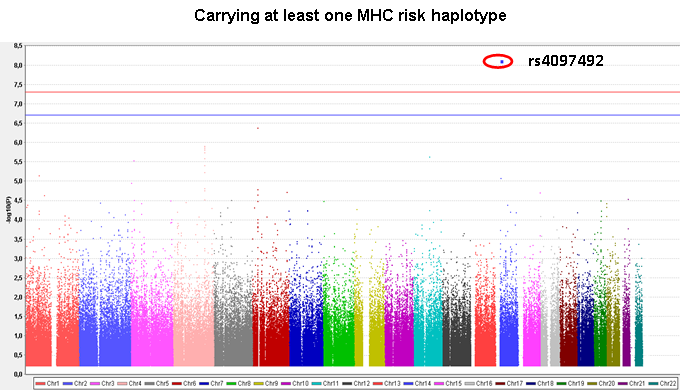
**

**Supplementary Figure 2b**

**
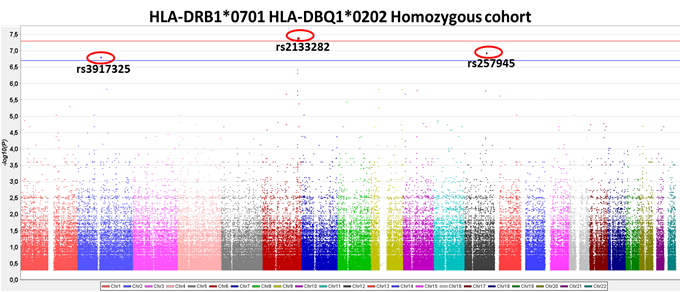
**

**Supplementary Figure 2c**

**
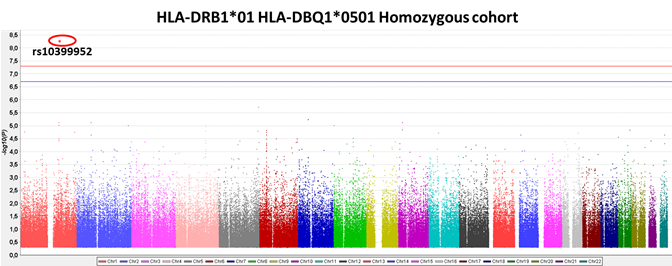
**

**Supplementary Figure 2d**

**
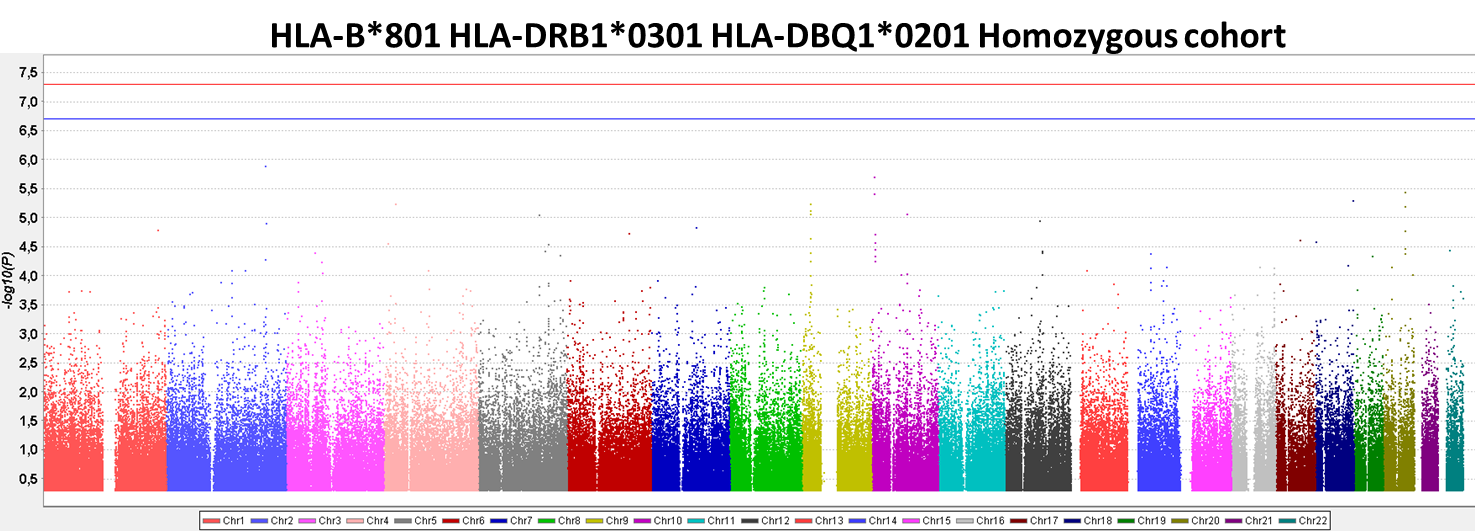
**

**Supplementary Figure 2e**

**
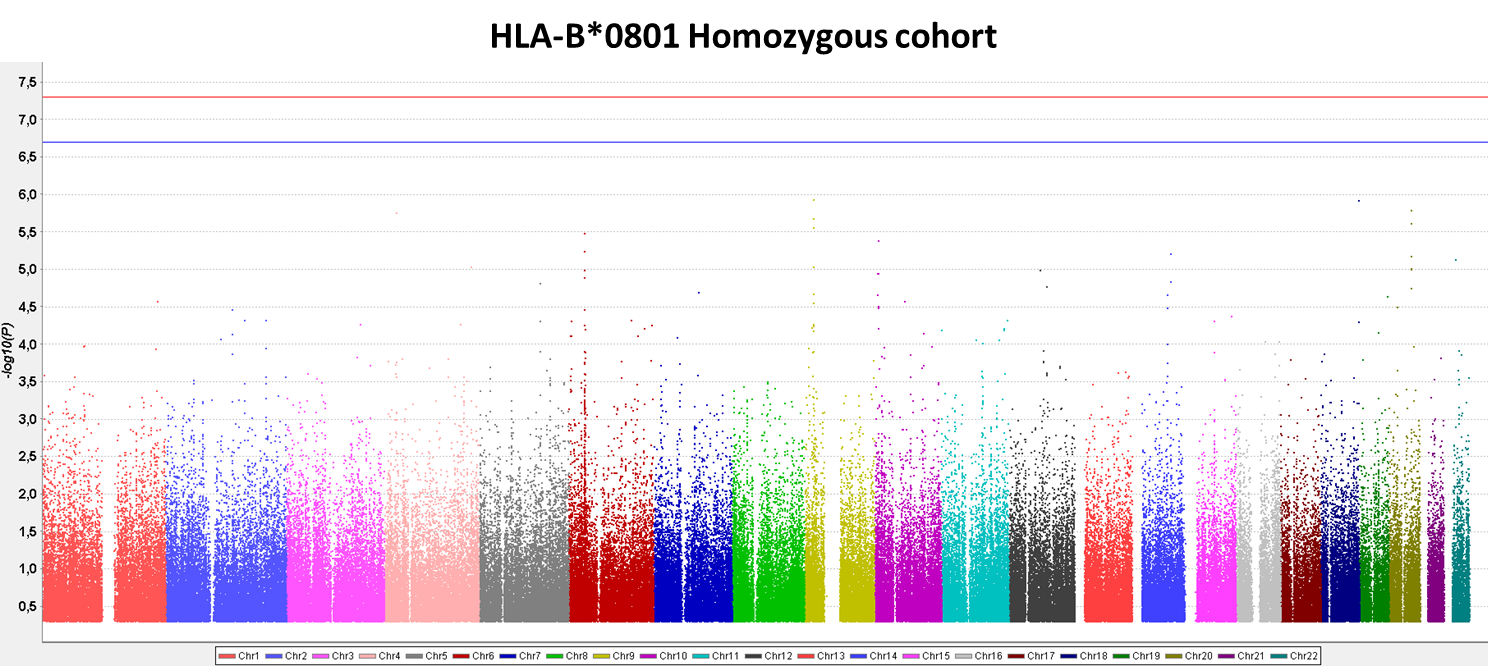
**

**Supplementary Figure 2f**

**
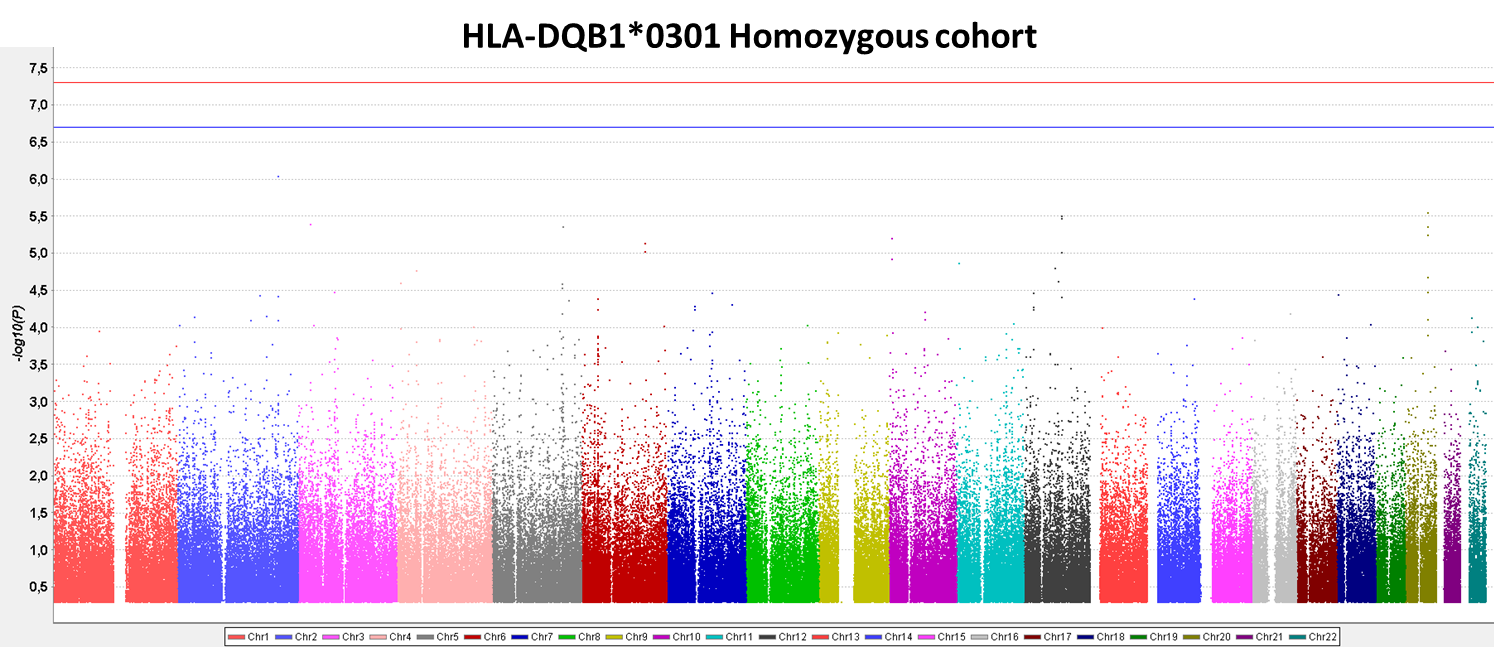
**

**Supplementary Figure 2g**

**
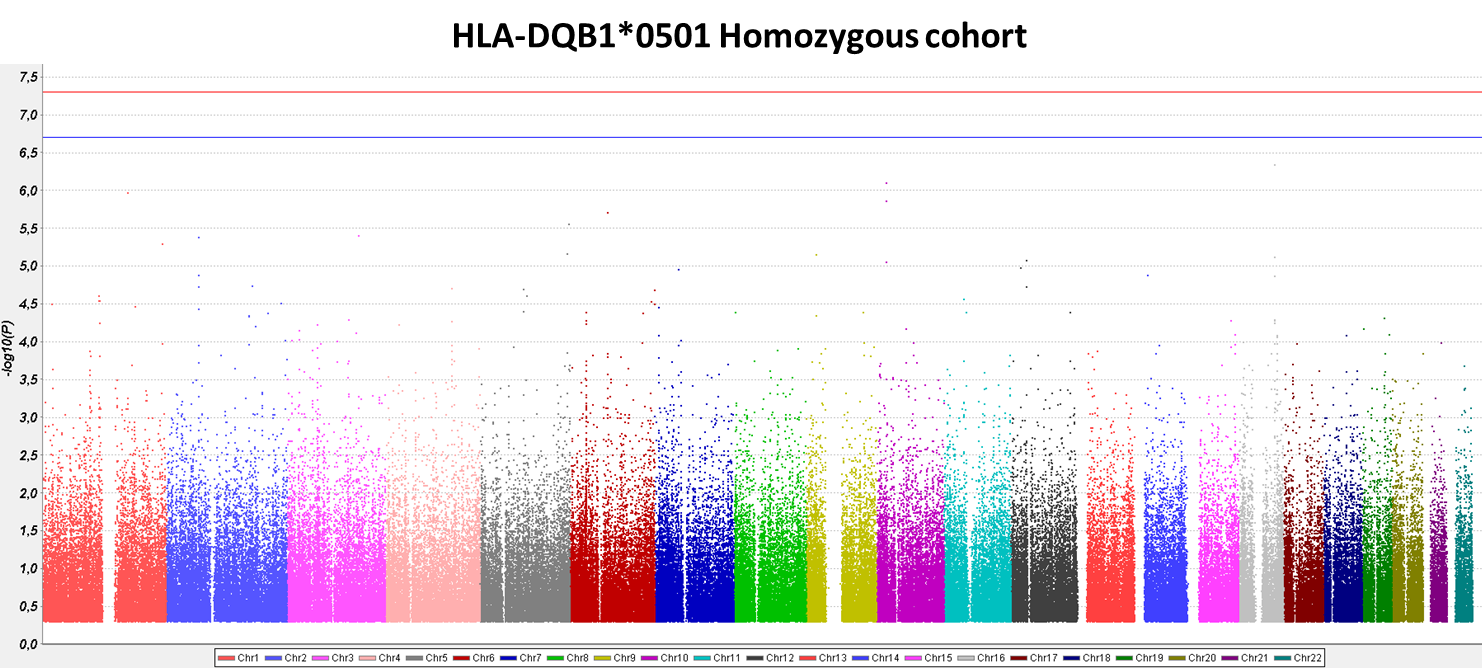
**

**Supplementary Figure 3.**


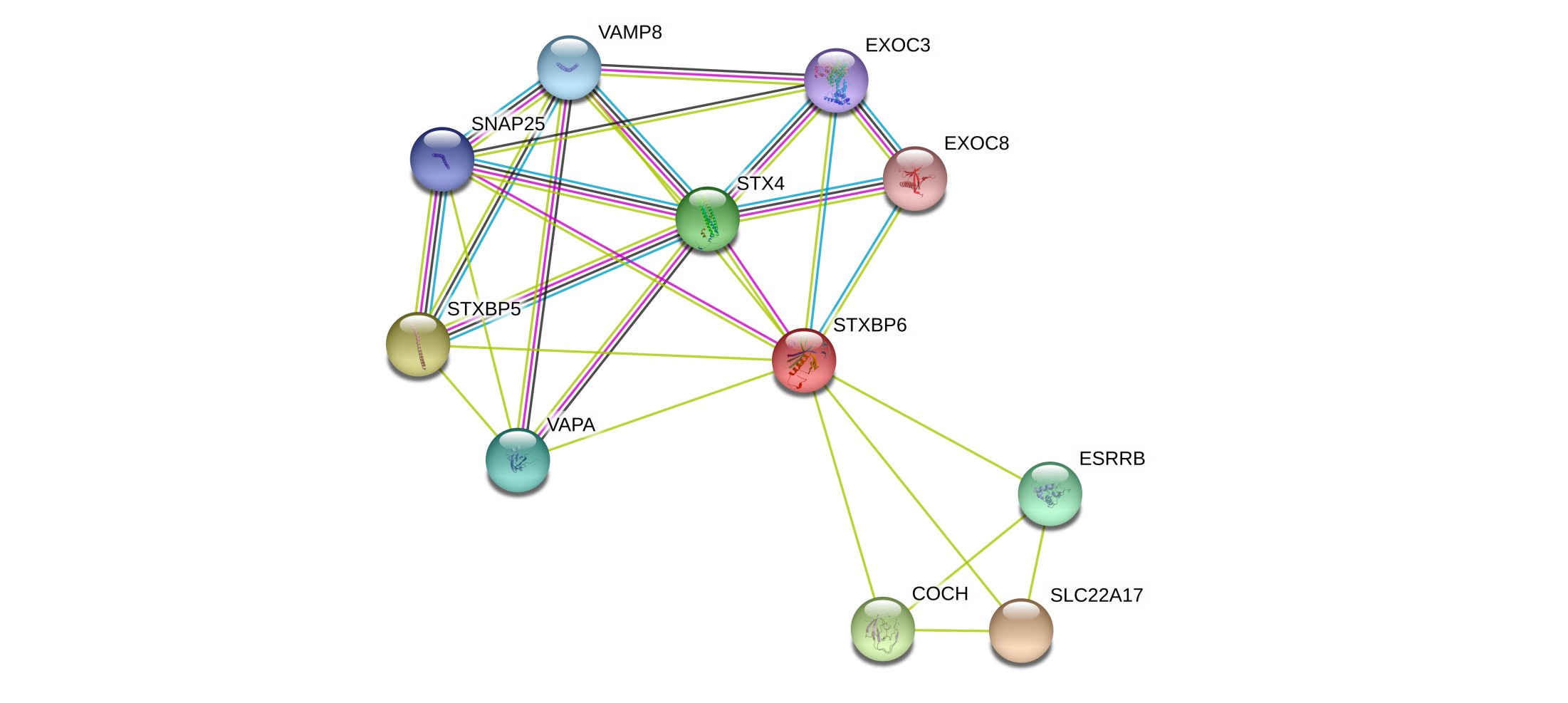

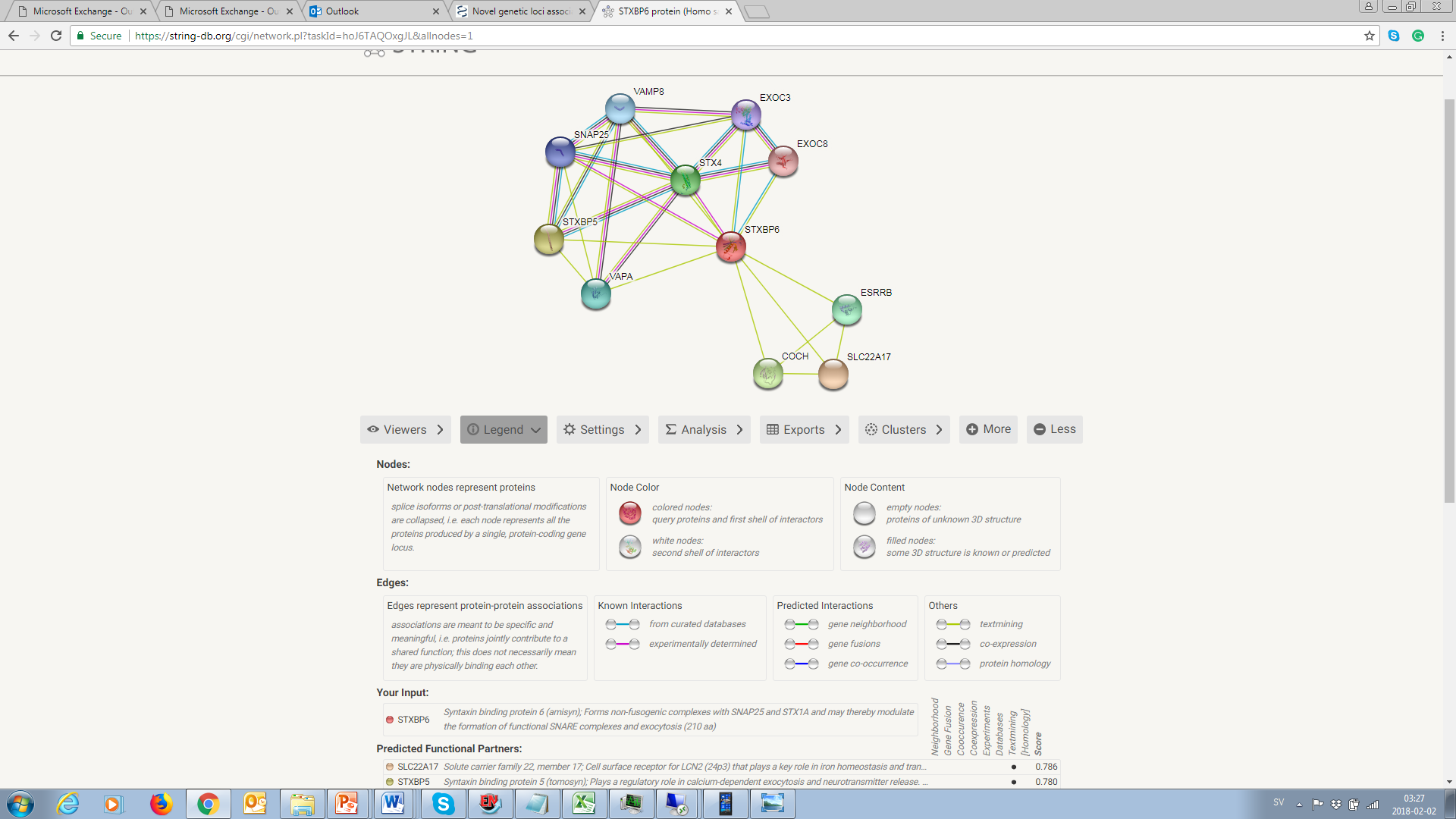

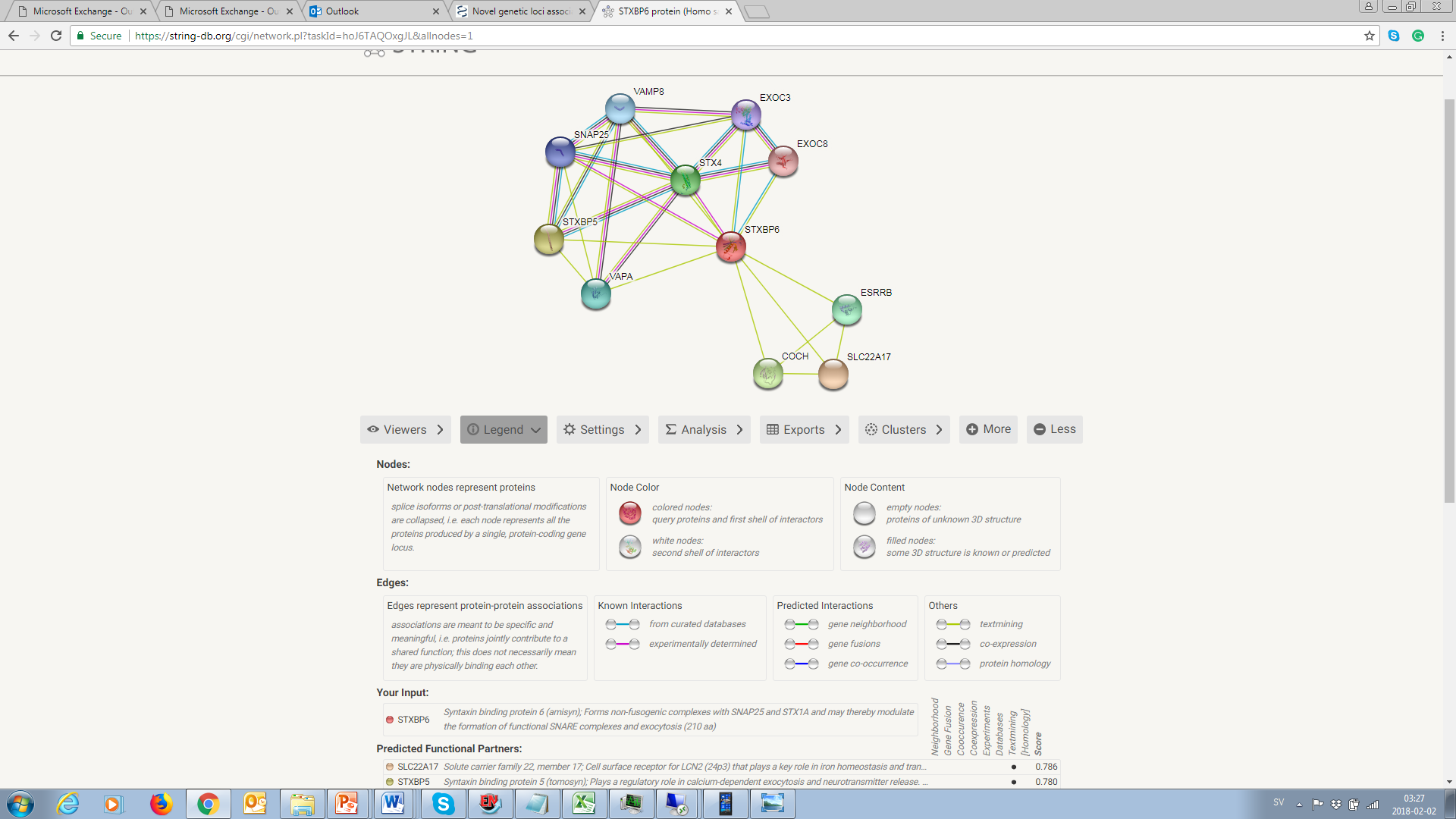

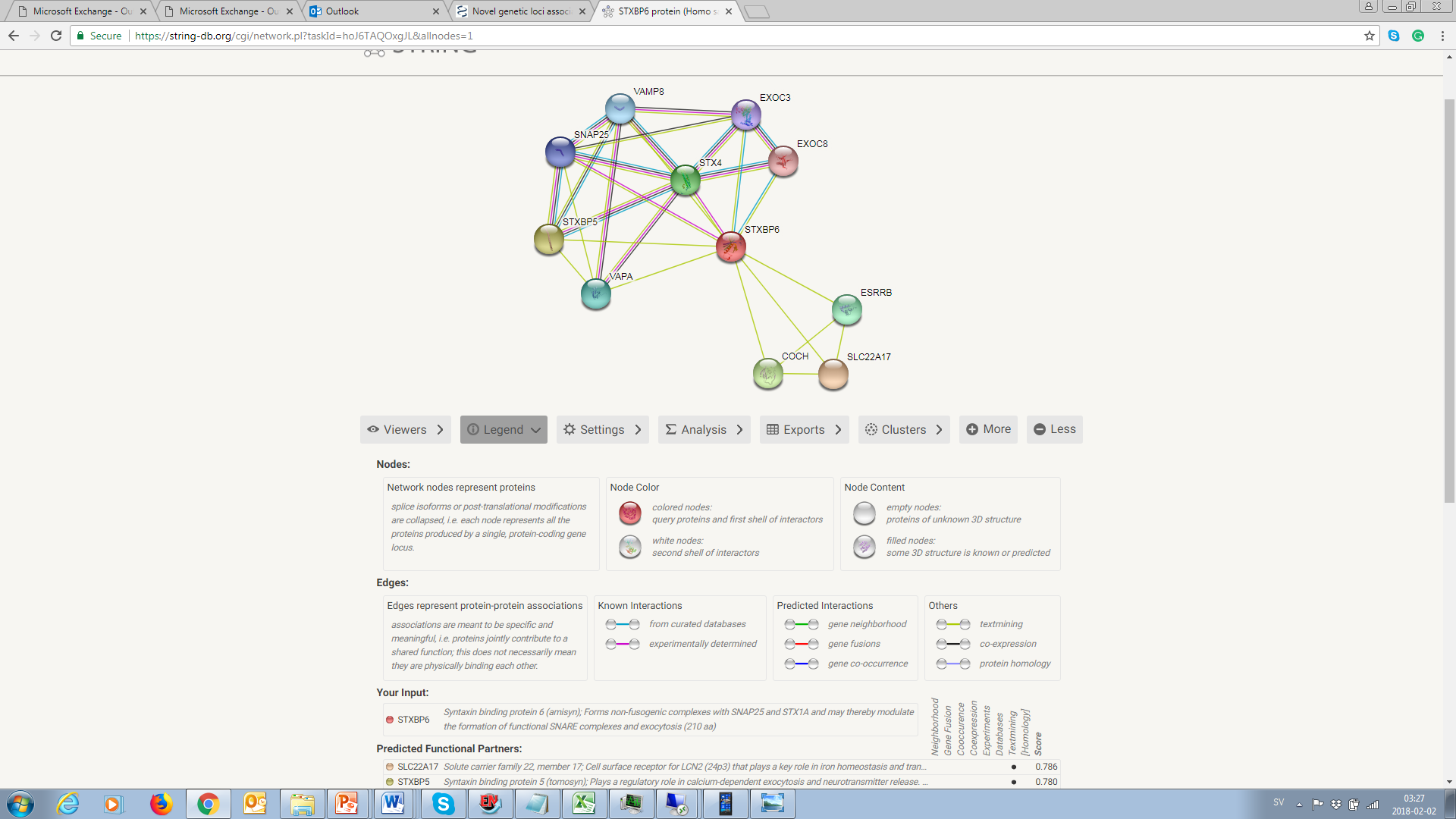


**Supplementary Figure 4a**

**
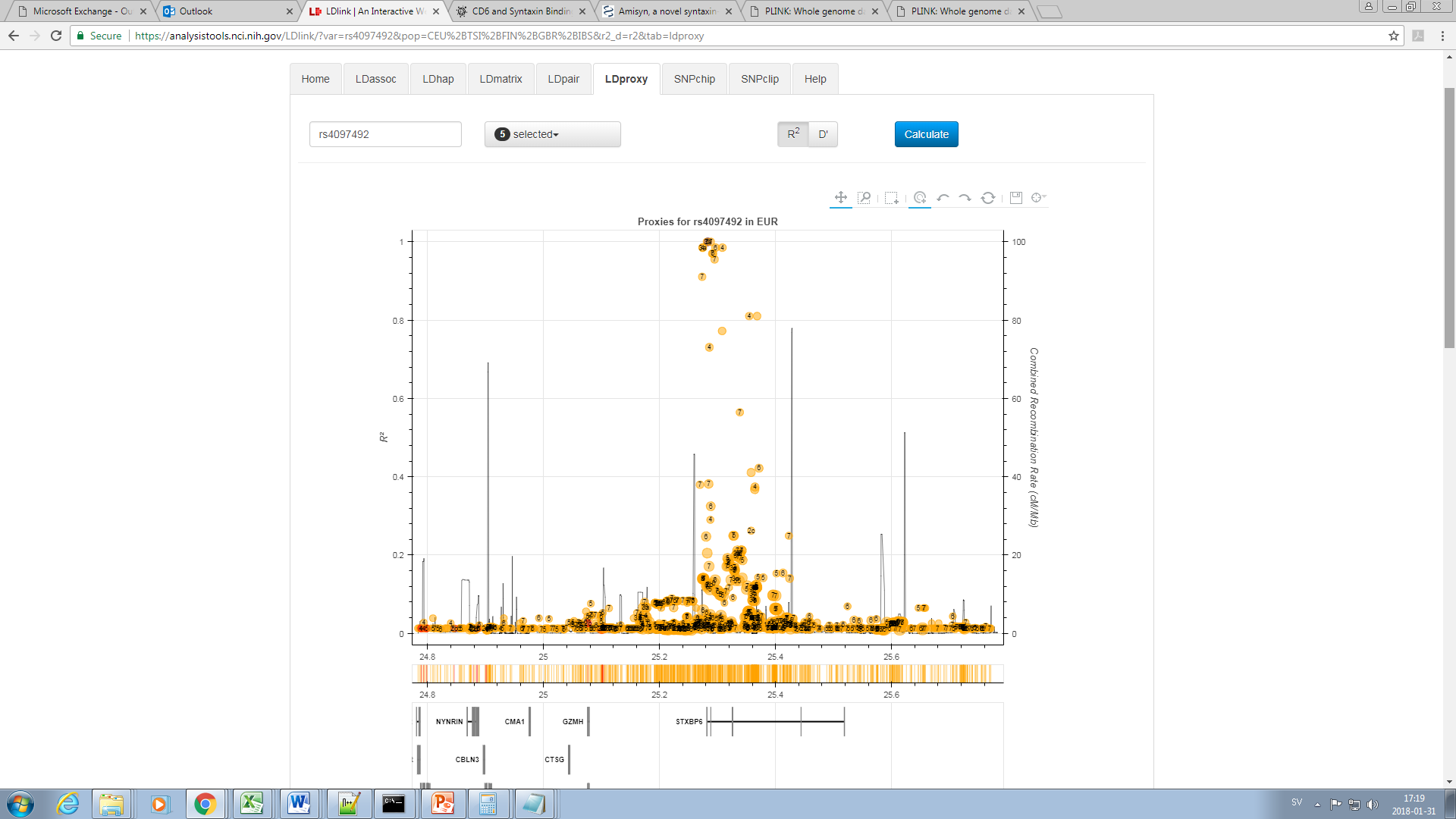
**

**Supplementary Figure 4b**

**
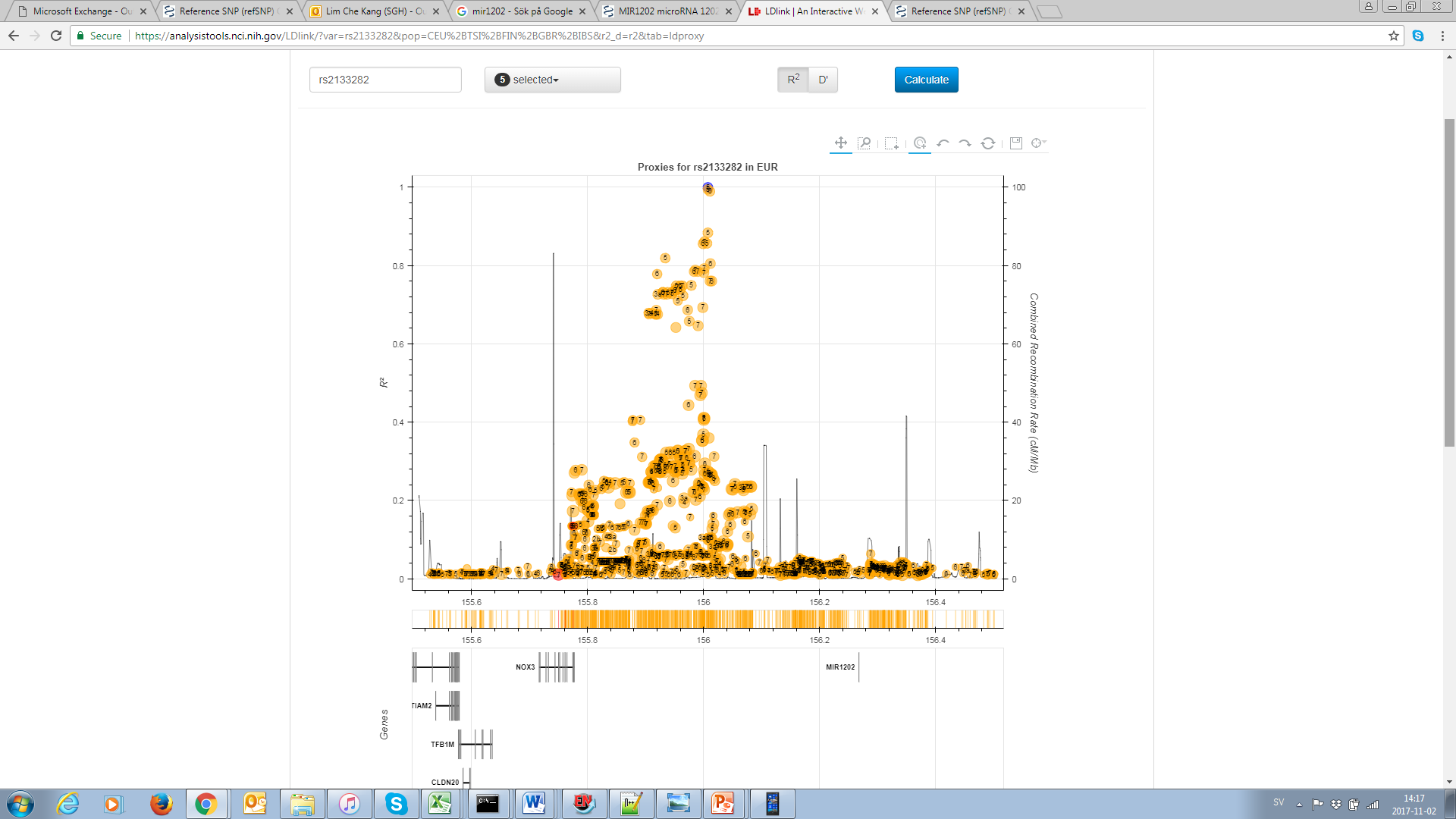
**

**Supplementary Figure 4c**

**
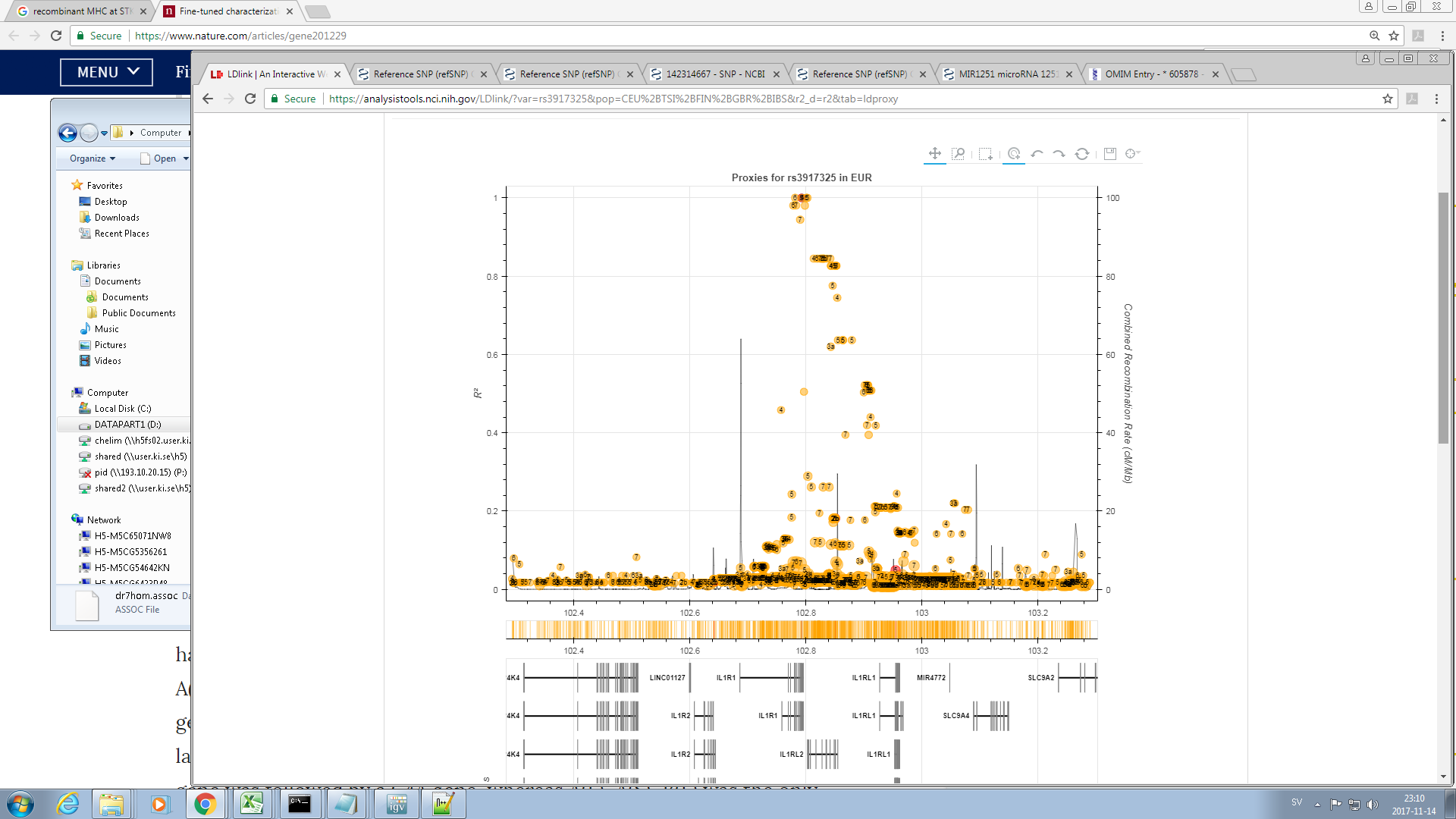
**

**Supplementary Figure 4d**

**
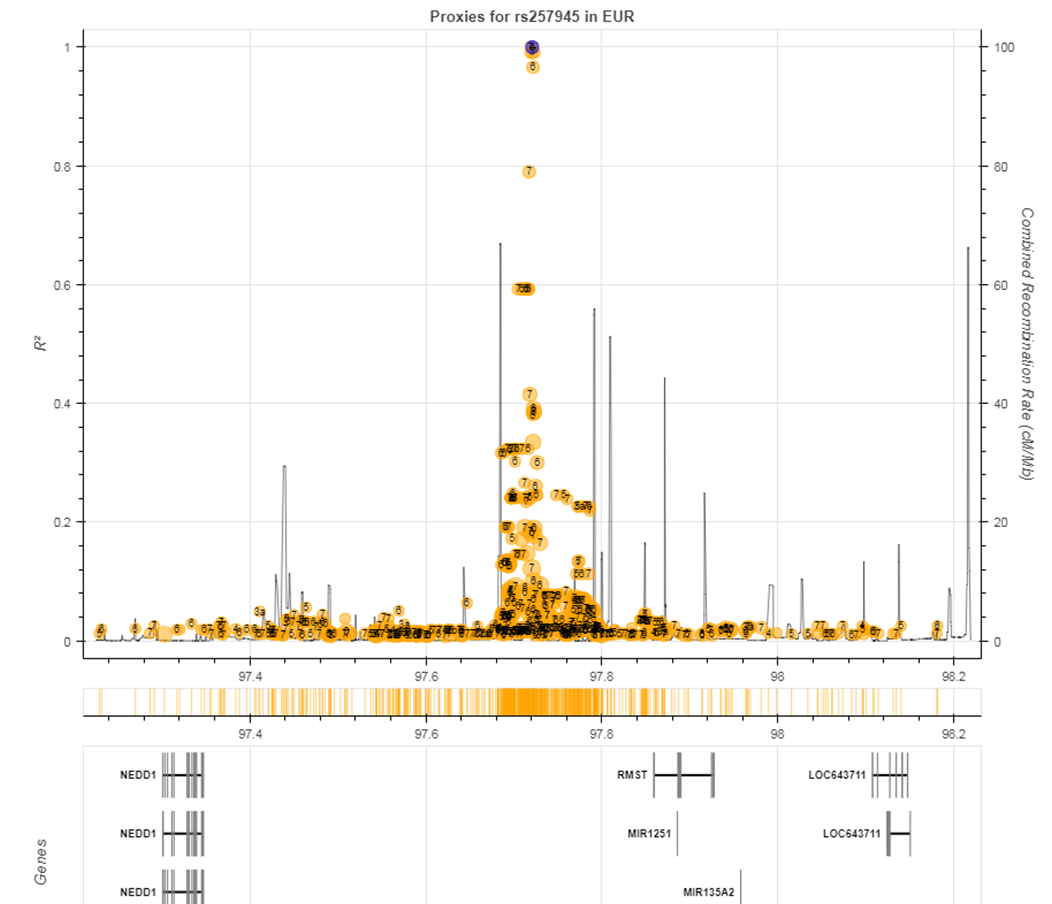
**

**Supplementary Figure 4e**

**
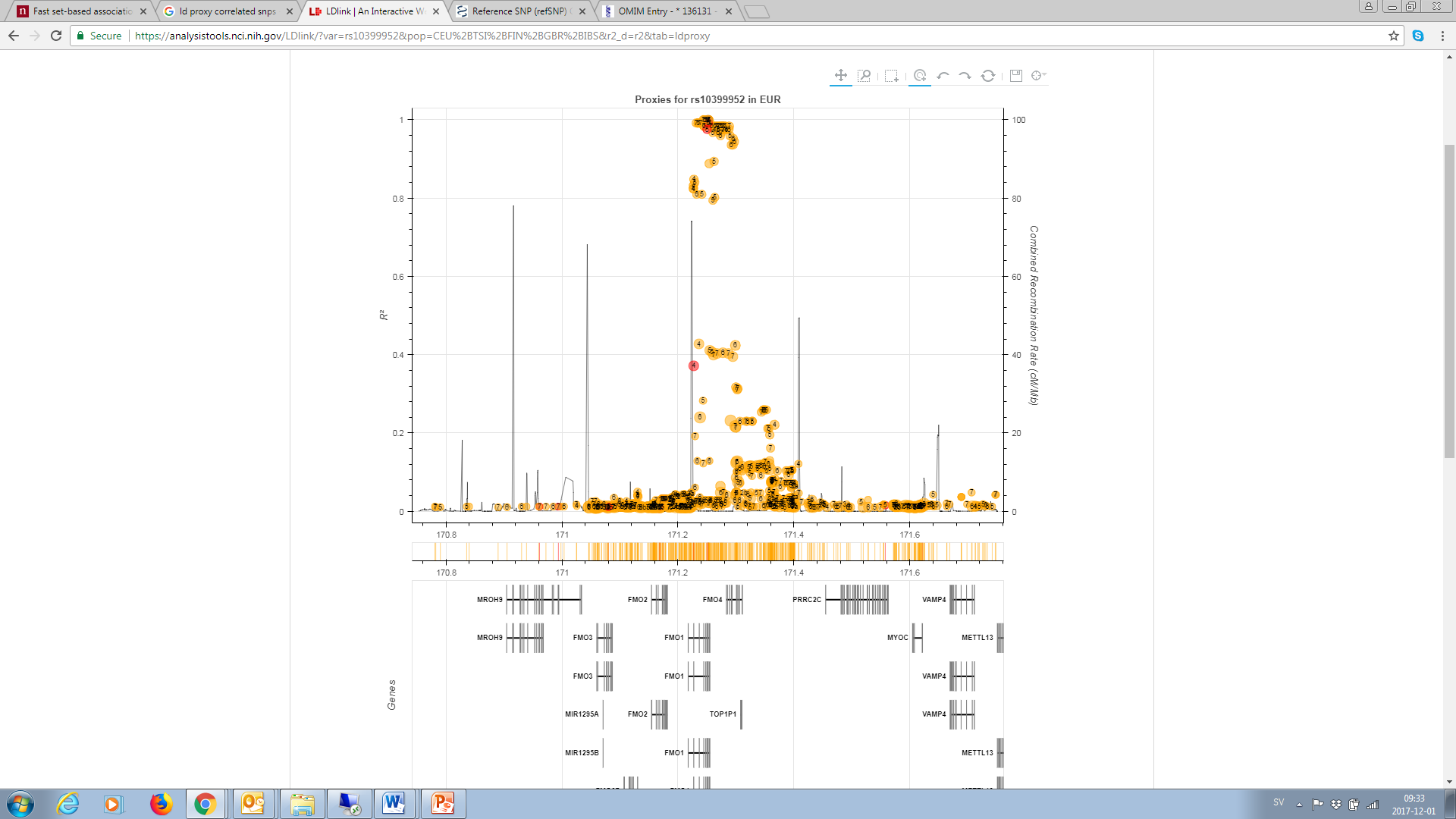
**

**Supplementary Figure 5**

**
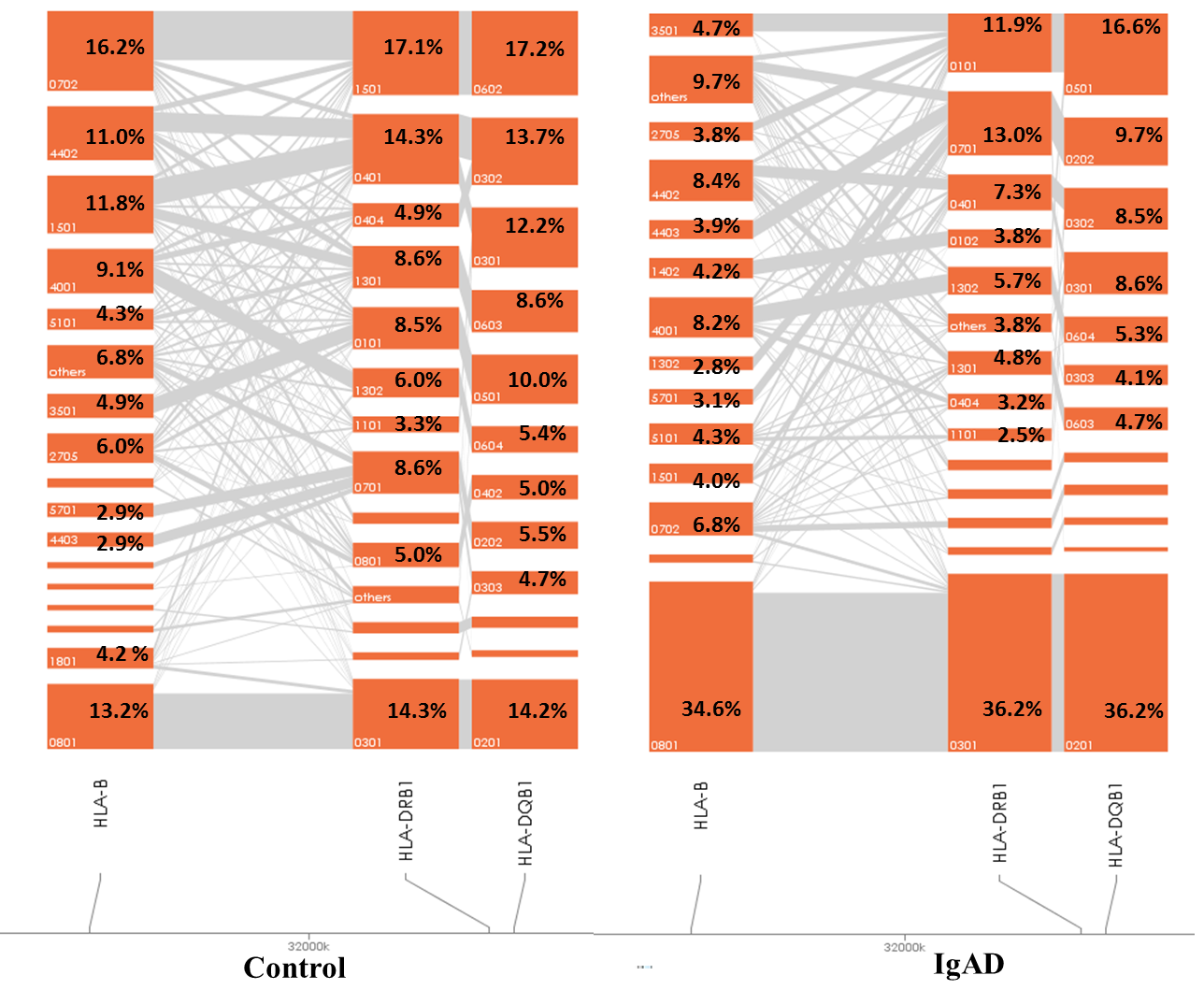
**

**Supplementary Table 1. Gene-based association analysis in subgroups carrying different MHC risk alleles**

| **Group** | **Gene** | **Chr** | **Bp Start** | **Bp End** | **No. SNPs** | **P** | **Immune function** | **Autoimmune disease association** |
| --- | --- | --- | --- | --- | --- | --- | --- | --- |
| *HLA-B*8:01 DRB1*03:01 DQB1*02:01* | *CD40* | 20 | 44746905 | 44758384 | 29 | 6.9 E-05* | Yes^1,2,3,4^ | SLE, RA, MS,IBD,T1D [^1-3^](#_ENREF_1) |
| *HLA-DRB1*01 DQB1*05:01* | *DHX38* | 16 | 72127614 | 72146811 | 14 | 8.6 E-05* | Indirect link with class switching [^4^](#_ENREF_4) | -- |
| Without Susceptibility Haplotype | *SFPQ* | 1 | 35649200 | 35658743 | 10 | 1.0 E-04* | Plays a role in NHEJ, Non-NHEJ and V(D)J recombination [^5^](#_ENREF_5) | -- |
|  | *TFAP2E* | 1 | 36038970 | 36060927 | 5 | 7.0 E-05* | -- | -- |
|  | *ZMYM4* | 1 | 35734567 | 35887545 | 9 | 1.1 E-04* | -- | Systemic autoimmune rheumatic diseases [^6^](#_ENREF_6) |
|  | *GIMAP5* | 7 | 150434435 | 150440737 | 28 | 1.9 E-04* | Yes^1,3^ | T1D,IBD,SLE,allergy,asthma [^7^](#_ENREF_7) |
|  | *B3GNT6* | 11 | 76745384 | 76753005 | 12 | 2.1 E-06** | Yes^1^ | Inflammatory colitis ^[8](#_ENREF_8)^ |
|  | *OXA1L* | 14 | 23235730 | 23240998 | 43 | 1.2 E-04* |  | Asthma ^[9](#_ENREF_9)^ |
|  | *TNFRSF13B* | 17 | 16842397 | 16875402 | 32 | 1.1 E-04* | Yes^1,2,3,4^ | CVID,IgAD ([49](#_ENREF_49), [50](#_ENREF_50)) |

** Significantly associated *P*_Gene_ <2.1 x10^-6^ ; * suggestive evidence for association *P*_Gene_ <2.1 x10^-4^

1. Immunogenetic Related Information Source (IRIS) [^10^](#_ENREF_10)
2. Immunome Database [^11^](#_ENREF_11)
3. The Immunology Database and Analysis Portal (Immport) [^12^](#_ENREF_12)
4. InnateDB [^13^](#_ENREF_13)

**Supplementary Table 2. Gene based analysis results in selected autoimmune diseases and IgAD associated genes in patients carrying different MHC risk alleles**

**
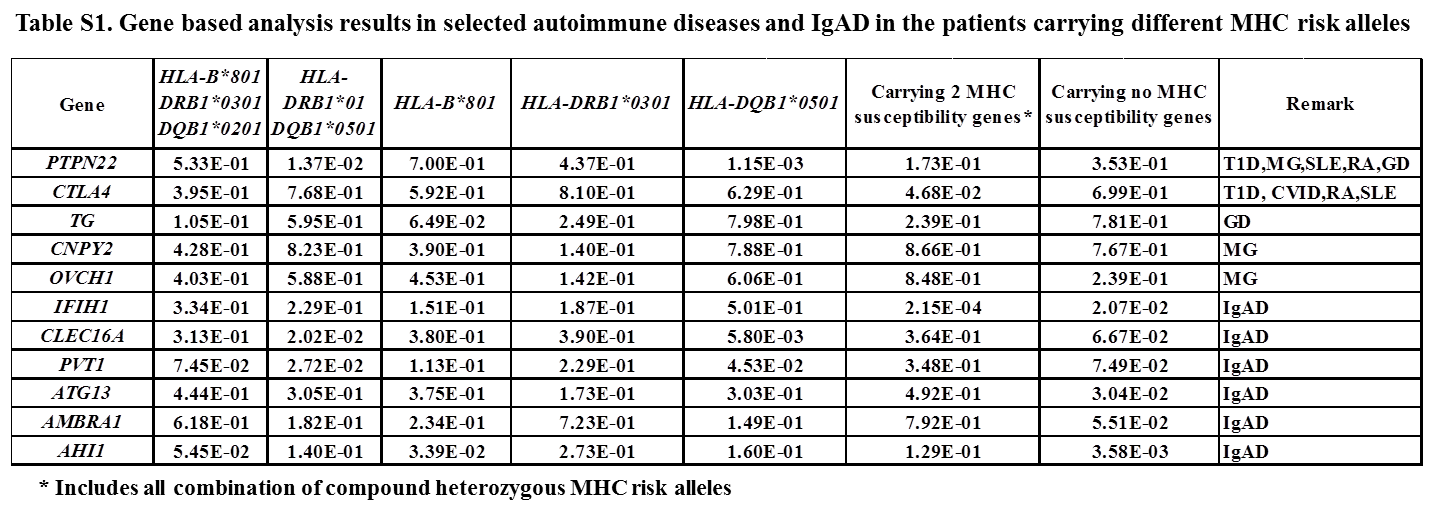
**

**Supplementary Table 3. Random IgAD disease cohort analysis and Permutations test in patients carrying at least one MHC risk alleles**

| **Random test using 400 IgAD samples** | | | | |
| --- | --- | --- | --- | --- |
| N_IgAD_ = 400 random samples |  |  |  |  |
|  | No | Chr | Reference SNP cluster ID | P |
| 400_R_test1 | 1 | 5 | rs7733250 | 8.32E-08 |
|  | 2 | 14 | rs4097492* | 1.32E-07 |
| 400_R_test2 | 1 | 14 | rs4097492* | 9.60E-10 |
| 400_R_test3 | 1 | 14 | rs4097492* | 4.32E-08 |
| 400_R_test4 | 1 | 14 | rs4097492* | 1.90E-08 |
|  | 2 | 4 | rs7658280 | 1.61E-07 |
| 400_R_test5 | 1 | 2 | rs6739613 | 7.96E-08 |
|  | 2 | 14 | rs4097492* | 1.72E-07 |
| **Random test using 500 IgAD samples** | | | | |
| N_IgAD_ = 500 random samples |  |  |  |  |
|  | No | Chr | Reference SNP cluster ID | P |
| 500_R_test1 | 1 | 14 | rs4097492* | 6.31E-08 |
| 500_R_test2 | 1 | 14 | rs4097492* | 1.05E-07 |
| 500_R_test3 | 1 | 14 | rs4097492* | 5.44E-10 |
| 500_R_test4 | 1 | 14 | rs4097492* | 1.22E-10 |
|  | 2 | 14 | rs17185348 | 4.63E-07 |
| 500_R_test5 | 1 | 14 | rs4097492* | 2.02E-08 |
|  | 2 | 4 | rs2714886 | 1.83E-07 |
|  | 3 | 4 | rs4571308 | 1.96E-07 |

Only P < 2.00 E-07 are listed

* The top SNP identified in this study

| **Permutation test using all IgAD samples** | | | | | |
| --- | --- | --- | --- | --- | --- |
| All samples (N_IgAD_ = 636) | P | P_perm1_ | P_perm2_ | P_perm3_ | P_perm4_ |
|  | 7.63E-09 | 1.00E-04 | 1.00E-05 | 1.00E-06 | 8.00E-08 |

Only top peak, rs4097492 result listed

Perm1: Empirical P value for permutations N= 10,000

Perm2: Empirical P value for permutations N= 100,000

Perm3: Empirical P value for permutations N= 1,000,000

Perm4: Empirical P value for permutations N= 100,000,000

**Reference for Supplementray Table 1**

1. van der Linden, M.P. *et al.* Association of a single-nucleotide polymorphism in CD40 with the rate of joint destruction in rheumatoid arthritis. *Arthritis Rheum* **60**, 2242-7 (2009).

2. Australia & New Zealand Multiple Sclerosis Genetics, C. Genome-wide association study identifies new multiple sclerosis susceptibility loci on chromosomes 12 and 20. *Nat Genet* **41**, 824-8 (2009).

3. Park, J.H. *et al.* Association analysis of CD40 polymorphisms with asthma and the level of serum total IgE. *Am J Respir Crit Care Med* **175**, 775-82 (2007).

4. Han, S., Park, J. & Lee, D.H. Protein DHX38 is a novel inhibitor of protein phosphatase 4. *Animal Cells and Systems* **19**, 236-244 (2015).

5. Rajesh, C., Baker, D.K., Pierce, A.J. & Pittman, D.L. The splicing-factor related protein SFPQ/PSF interacts with RAD51D and is necessary for homology-directed repair and sister chromatid cohesion. *Nucleic Acids Res* **39**, 132-45 (2011).

6. Hudson, M. *et al.* Novel insights into systemic autoimmune rheumatic diseases using shared molecular signatures and an integrative analysis. *Epigenetics* **12**, 433-440 (2017).

7. Heinonen, M.T. *et al.* GIMAP GTPase family genes: potential modifiers in autoimmune diabetes, asthma, and allergy. *J Immunol* **194**, 5885-94 (2015).

8. An, G. *et al.* Increased susceptibility to colitis and colorectal tumors in mice lacking core 3-derived O-glycans. *J Exp Med* **204**, 1417-29 (2007).

9. Costa, G.N. *et al.* A genome-wide association study of asthma symptoms in Latin American children. *BMC Genet* **16**, 141 (2015).

10. Kelley, J., de Bono, B. & Trowsdale, J. IRIS: a database surveying known human immune system genes. *Genomics* **85**, 503-11 (2005).

11. Ortutay, C., Siermala, M. & Vihinen, M. Molecular characterization of the immune system: emergence of proteins, processes, and domains. *Immunogenetics* **59**, 333-48 (2007).

12. Bhattacharya, S. *et al.* ImmPort: disseminating data to the public for the future of immunology. *Immunol Res* **58**, 234-9 (2014).

13. Breuer, K. *et al.* InnateDB: systems biology of innate immunity and beyond--recent updates and continuing curation. *Nucleic Acids Res* **41**, D1228-33 (2013).
